# Social Relationships and Mortality Risk: A Meta-analytic Review

**DOI:** 10.1371/journal.pmed.1000316

**Published:** 2010-07-27

**Authors:** Julianne Holt-Lunstad, Timothy B. Smith, J. Bradley Layton

**Affiliations:** 1 Department of Psychology, Brigham Young University, Provo, Utah, United States of America; 2 Department of Counseling Psychology, Brigham Young University, Provo, Utah, United States of America; 3 Department of Epidemiology, University of North Carolina at Chapel Hill, Chapel Hill, North Carolina, United States of America; University of Cambridge, United Kingdom

## Abstract

**Background:**

The quality and quantity of individuals' social relationships has been linked not only to mental health but also to both morbidity and mortality.

**Objectives:**

This meta-analytic review was conducted to determine the extent to which social relationships influence risk for mortality, which aspects of social relationships are most highly predictive, and which factors may moderate the risk.

**Data Extraction:**

Data were extracted on several participant characteristics, including cause of mortality, initial health status, and pre-existing health conditions, as well as on study characteristics, including length of follow-up and type of assessment of social relationships.

**Results:**

Across 148 studies (308,849 participants), the random effects weighted average effect size was OR = 1.50 (95% CI 1.42 to 1.59), indicating a 50% increased likelihood of survival for participants with stronger social relationships. This finding remained consistent across age, sex, initial health status, cause of death, and follow-up period. Significant differences were found across the type of social measurement evaluated (*p*<0.001); the association was strongest for complex measures of social integration (OR = 1.91; 95% CI 1.63 to 2.23) and lowest for binary indicators of residential status (living alone versus with others) (OR = 1.19; 95% CI 0.99 to 1.44).

**Conclusions:**

The influence of social relationships on risk for mortality is comparable with well-established risk factors for mortality.

*Please see later in the article for the Editors' Summary*

## Abstract 2

In a meta-analysis, Julianne Holt-Lunstad and colleagues find that individuals' social relationships have as much influence on mortality risk as other well-established risk factors for mortality, such as smoking.

## Editors' Summary

**Background:**

Humans are naturally social. Yet, the modern way of life in industrialized countries is greatly reducing the quantity and quality of social relationships. Many people in these countries no longer live in extended families or even near each other. Instead, they often live on the other side of the country or even across the world from their relatives. Many also delay getting married and having children. Likwise, more and more people of all ages in developed countries are living alone, and loneliness is becoming increasingly common. In the UK, according to a recent survey by the Mental Health Foundation, 10% of people often feel lonely, a third have a close friend or relative who they think is very lonely, and half think that people are getting lonelier in general. Similarly, across the Atlantic, over the past two decades there has been a three-fold increase in the number of Americans who say they have no close confidants. There is reason to believe that people are becoming more socially isolated.

**Why Was This Study Done?:**

Some experts think that social isolation is bad for human health. They point to a 1988 review of five prospective studies (investigations in which the characteristics of a population are determined and then the population is followed to see whether any of these characteristics are associated with specific outcomes) that showed that people with fewer social relationships die earlier on average than those with more social relationships. But, even though many prospective studies of mortality (death) have included measures of social relationships since that first review, the idea that a lack of social relationships is a risk factor for death is still not widely recognized by health organizations and the public. In this study, therefore, the researchers undertake a systematic review and meta-analysis of the relevant literature to determine the extent to which social relationships influence mortality risk and which aspects of social relationships are most predictive of mortality. A systematic review uses predefined criteria to identify all the research on a given topic; a meta-analysis uses statistical methods to combine the results of several studies.

**What Did the Researchers Do and Find?:**

The researchers identified 148 prospective studies that provided data on individuals' mortality as a function of social relationships and extracted an “effect size” from each study. An effect size quantifies the size of a difference between two groups—here, the difference in the likelihood of death between groups that differ in terms of their social relationships. The researchers then used a statistical method called “random effects modeling” to calculate the average effect size of the studies expressed as an odds ratio (OR)—the ratio of the chances of an event happening in one group to the chances of the same event happening in the second group. They report that the average OR was 1.5. That is, people with stronger social relationships had a 50% increased likelihood of survival than those with weaker social relationships. Put another way, an OR of 1.5 means that by the time half of a hypothetical sample of 100 people has died, there will be five more people alive with stronger social relationships than people with weaker social relationships. Importantly, the researchers also report that social relationships were more predictive of the risk of death in studies that considered complex measurements of social integration than in studies that considered simple evaluations such as marital status.

**What Do These Findings Mean?:**

These findings indicate that the influence of social relationships on the risk of death are comparable with well-established risk factors for mortality such as smoking and alcohol consumption and exceed the influence of other risk factors such as physical inactivity and obesity. Furthermore, the overall effect of social relationships on mortality reported in this meta-analysis might be an underestimate, because many of the studies used simple single-item measures of social isolation rather than a complex measurement. Although further research is needed to determine exactly how social relationships can be used to reduce mortality risk, physicians, health professionals, educators, and the media should now acknowledge that social relationships influence the health outcomes of adults and should take social relationships as seriously as other risk factors that affect mortality, the researchers conclude.

**Additional Information:**

Please access these Web sites via the online version of this summary at http://dx.doi.org/10.1371/journal.pmed.1000316.

- The Mental Health America Live Your Life Well page includes information about how social relationships improve both mental and physical health
- The Mental Health Foundation, a UK charity, has information on loneliness and mental health; its report “The Lonely Society?” can be downloaded from this page
- The Mayo Clinic has information on social support as a way to manage stress
- The Pew Research Foundation has information on technology and social isolation
- Wikipedia has a page on social isolation (note that Wikipedia is a free online encyclopedia that anyone can edit; available in several languages)

## Introduction

*“Social relationships, or the relative lack thereof, constitute a major risk factor for health—rivaling the effect of well established health risk factors such as cigarette smoking, blood pressure, blood lipids, obesity and physical activity”*

—House, Landis, and Umberson; *Science* 1988 [1]

Two decades ago a causal association between social relationships and mortality was proposed after a review of five large prospective studies concluded that social relationships predict mortality [1]. Following the publication of this provocative review, the number of prospective studies of mortality that included measures of social relationships increased exponentially. Although the inverse association between social relationships and nonsuicide mortality has received increased attention in research, neither major health organizations nor the general public recognize it as a risk factor for mortality. This may be due in part to the fact that the literature has become unwieldy, with wide variation in how social relationships are measured across a large number of studies and disappointing clinical trials [2]. “Social relationships” has perhaps become viewed as a fuzzy variable, lacking the level of precision and control that is preferred in biomedical research. Thus, the large corpus of relevant empirical research is in need of synthesis and refinement.

Current evidence also indicates that the quantity and/or quality of social relationships in industrialized societies are decreasing. For instance, trends reveal reduced intergenerational living, greater social mobility, delayed marriage, dual-career families, increased single-residence households, and increased age-related disabilities [3],[4]. More specifically, over the last two decades there has been a three-fold increase in the number of Americans who report having no confidant—now the modal response [3]. Such findings suggest that despite increases in technology and globalization that would presumably foster social connections, people are becoming increasingly more socially isolated. Given these trends, understanding the nature and extent of the association between social relationships and mortality is of increased temporal importance.

There are two general theoretical models that propose processes through which social relationships may influence health: the stress buffering and main effects models [5]. The buffering hypothesis suggests that social relationships may provide resources (informational, emotional, or tangible) that promote adaptive behavioral or neuroendocrine responses to acute or chronic stressors (e.g., illness, life events, life transitions). The aid from social relationships thereby moderates or *buffers* the deleterious influence of stressors on health. From this perspective, the term *social support* is used to refer to the real or perceived availability of social resources [6]. The main effects model proposes that social relationships may be associated with protective health effects through more direct means, such as cognitive, emotional, behavioral, and biological influences that are not explicitly intended as help or support. For instance, social relationships may directly encourage or indirectly model healthy behaviors; thus, being part of a social network is typically associated with conformity to social norms relevant to health and self-care. In addition, being part of a social network gives individuals meaningful roles that provide self-esteem and purpose to life [7],[8].

Social relationships have been defined and measured in diverse ways across studies. Despite striking differences, three major components of social relationships are consistently evaluated [5]: (a) the degree of integration in social networks [9], (b) the social interactions that are intended to be supportive (i.e., received social support), and (c) the beliefs and perceptions of support availability held by the individual (i.e., perceived social support). The first subconstruct represents the structural aspects of social relationships and the latter two represent the functional aspects. Notably, these different subconstructs are only moderately intercorrelated, typically ranging between *r* = 0.20 and 0.30 [9],[10]. While all three components have been shown to be associated with morbidity and mortality, it is thought that each may influence health in different ways [11],[12]. Because it is presently unclear whether any single aspect of social relationships is more predictive than others, synthesis of data across studies using several types of measures of social relationships would allow for essential comparisons that have not been conducted on such a large scale.

Empirical data suggest the medical relevance of social relationships in improving patient care [13], increasing compliance with medical regimens [13], and promoting decreased length of hospitalization [14],[15]. Likewise, social relationships have been linked to the development [16],[17] and progression [18]–[21] of cardiovascular disease [22]—a leading cause of death globally. Therefore, synthesis of the current empirical evidence linking social relationships and mortality, along with clarifications of potential moderators, may be particularly relevant to public health and clinical practice for informing interventions and policies aimed at reducing risk for mortality.

To address these issues, we conducted a meta-analysis of the literature investigating the association between social relationships and mortality. Specifically, we addressed the following questions: What is the overall magnitude of the association between social relationships and mortality across research studies? Do structural versus functional aspects of social relationships differentially impact the risk for mortality? Is the association moderated by participant characteristics (age, gender, health status, cause of mortality) or by study characteristics (length of clinical follow-up, inclusion of statistical controls)? Is the influence of social relationships on mortality a gradient or threshold effect?

## Methods

### Identification of Studies

To identify published and unpublished studies of the association between social relationships and mortality, we used three techniques. First, we conducted searches of studies from January 1900 to January 2007 using several electronic databases: Dissertation Abstracts, HealthSTAR, Medline, Mental Health Abstracts, PsycINFO, Social Sciences Abstracts, Sociological Abstracts via SocioFile, Academic Search Premier, ERIC, and Family & Society Studies Worldwide. To capture the broadest possible sample of relevant articles, we used multiple search terms, including *mortality*, *death*, *decease(d)*, *died*, *dead*, and *remain(ed) alive*, which were crossed with search words related to social relationships, including the terms *social* and *interpersonal* linked to the following words: *support*, *network*, *integration*, *participation*, *cohesion*, *relationship*, *capital*, *and isolation* To reduce inadvertent omissions, we searched databases yielding the most citations (Medline, PsycINFO) two additional times. Next, we manually examined the reference sections of past reviews and of studies meeting the inclusion criteria to locate articles not identified in the database searches. Finally, we sent solicitation letters to authors who had published three or more articles on the topic.

### Inclusion Criteria

**Figure 1:**
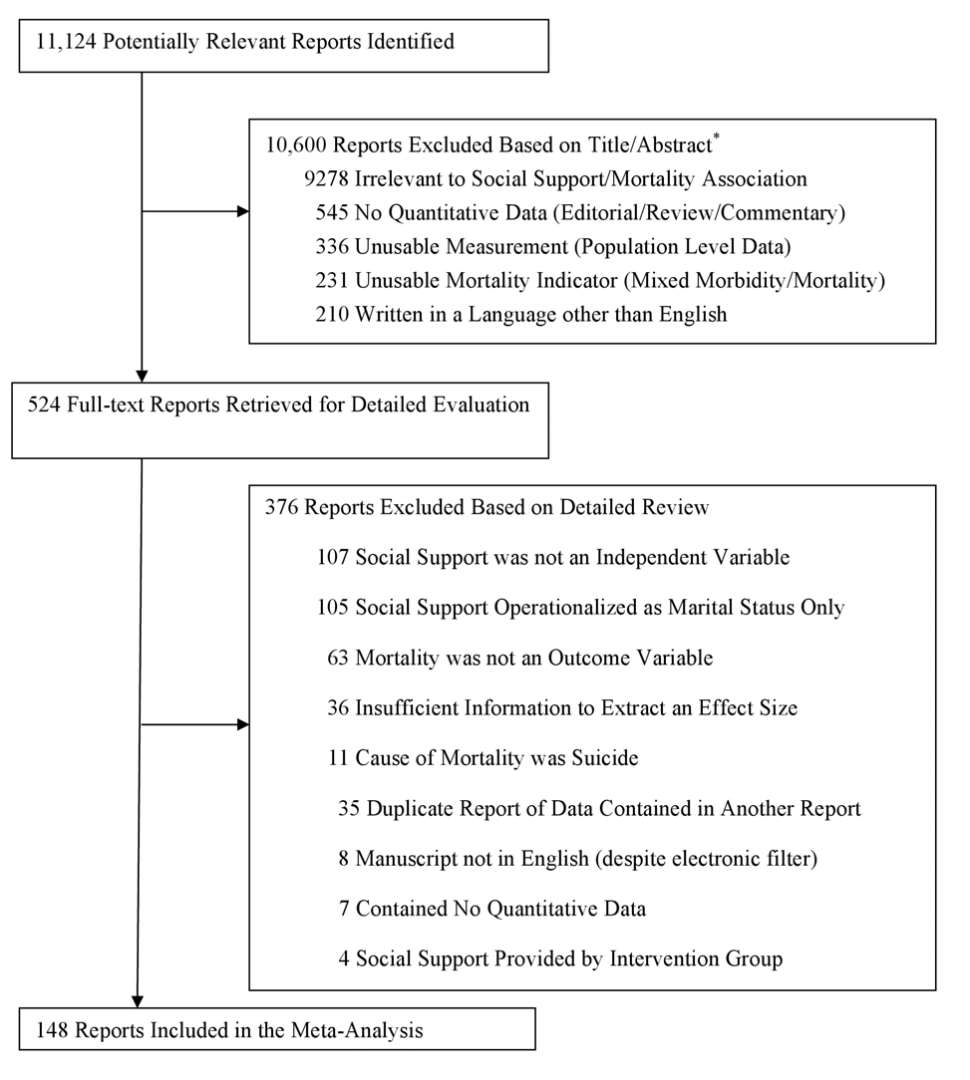
Flow diagram.

We included in the meta-analysis studies that provided quantitative data regarding individuals' mortality as a function of social relationships, including both structural and functional aspects [23]. Because we were interested in the impact of social relationships on disease, we excluded studies in which mortality was a result of suicide or injury. We also excluded studies in which the only measurement of social support was an intervention provided within the context of the study (e.g., support group), the source of social support was nonhuman (e.g., a pet or higher power), or the social support was provided to others (i.e., giving support to others or measures of others' benefit from the support provided) rather than to the individual tracked for mortality status. We coded studies that included participant marital status as one of several indicators of social support, but we excluded studies in which marital status was the only indicator of social support. We also excluded studies in which the outcome was not explicitly and solely mortality (e.g., combined outcomes of morbidity/mortality). Reports with exclusively aggregated data (e.g., census-level statistics) were also excluded. Manuscripts coded were all written in English, which accounted for 98% of the total retrieved. See Figure 1 for additional details.

### Data Abstraction

To increase the accuracy of coding and data entry, each article was initially coded by two raters. Subsequently, the same article was independently coded by two additional raters. Coders extracted several objectively verifiable characteristics of the studies: (a) the number of participants and their composition by age, gender, marital status, distress level, health status, and pre-existing health conditions (if any), as well as the percentage of smokers and percentage of physically active individuals, and, of course, the cause of mortality; (b) the length of follow up; (c) the research design; and (d) the aspect of social relationships evaluated.

Data within studies were often reported in terms of odds ratios (ORs), the likelihood of mortality across distinct levels of social relationships. Because OR values cannot be meaningfully aggregated, all effect sizes reported within studies were transformed to the natural log OR (lnOR) for analyses and then transformed back to OR for interpretation. When effect size data were reported in any metric other than OR or lnOR, we transformed those values using statistical software programs and macros (e.g., Comprehensive Meta-Analysis [24]). In some cases when direct statistical transformation proved impossible, we calculated the corresponding effect sizes from frequency data in matrices of mortality status by social relationship status. When frequency data were not reported, we recovered the cell probabilities from the reported ratio and marginal probabilities. When survival analyses (i.e., hazard ratios) were reported, we calculated the effect size from the associated level of statistical significance, often derived from 95% confidence intervals (CIs). Across all studies we assigned OR values less than 1.00 to data indicative of increased mortality and OR values greater than 1.00 to data indicative of decreased mortality for individuals with relatively higher levels of social relationships.

When multiple effect sizes were reported within a study at the same point in time (e.g., across different measures of social relationships), we averaged the several values (weighted by standard error) to avoid violating the assumption of independent samples. In such cases, the aggregate standard error value for the lnOR were estimated on the basis of the total frequency data without adjustment for possible correlation among the averaged values. Although this method was imprecise, the manuscripts included in the meta-analysis did not report the information necessary to make the statistical adjustments, and we decided not to impute values given the wide range possible. In analyzing the data we used the shifting units of analysis approach [25] which minimizes the threat of nonindependence in the data while at the same time allowing more detailed follow-up analyses to be conducted (i.e., examination of effect size heterogeneity).

When multiple reports contained data from the same participants (publications of the same database), we selected the report containing the whole sample and eliminated reports of subsamples. When multiple reports contained the same whole sample, we selected the one with the longest follow-up duration. When multiple reports with the same whole sample were of the same duration, we selected the one reporting the greatest number of measures of social relationships.

In cases where multiple effect sizes were reported across different levels of social relationships (i.e., high versus medium, medium versus low), we extracted the value with the greatest contrast (i.e., high versus low). When a study contained multiple effect sizes across time, we extracted the data from the longest follow-up period. If a study used statistical controls in calculating an effect size, we extracted the data from the model utilizing the fewest statistical controls so as to remain as consistent as possible across studies (and we recorded the type and number of covariates used within each study to run post hoc comparative analyses). We coded the research design used rather than estimate risk of individual study bias. The coding protocol is available from the authors.

The majority of information obtained from the studies was extracted verbatim from the reports. As a result, the inter-rater agreement was quite high for categorical variables (mean Cohen's *kappa* = 0.73, *SD* = 0.13) and for continuous variables (mean intraclass correlation [26] = 0.80, *SD* = .14). Discrepancies across coding pairs were resolved through further scrutiny of the manuscript until consensus was obtained.

Aggregate effect sizes were calculated using random effects models following confirmation of heterogeneity. A random effects approach produces results that generalize beyond the sample of studies actually reviewed [27]. The assumptions made in this meta-analysis clearly warrant this method: The belief that certain variables serve as moderators of the observed association between social relationships and mortality implies that the studies reviewed will estimate different population effect sizes. Random effects models take such between-studies variation into account, whereas fixed effects models do not [28]. In each analysis conducted, we examined the remaining variance to confirm that random effects models were appropriate.

## Results

Statistically nonredundant effect sizes were extracted from 148 studies ([29]–[176]; see Table 1). Data were reported from 308,849 participants, with 51% from North America, 37% from Europe, 11% from Asia, and 1% from Australia. Across all studies, the average age of participants at initial evaluation was 63.9 years, and participants were evenly represented across sex (49% female, 51% male). Of the studies examined, 60% involved community samples, but 24% examined individuals receiving outpatient medical treatment, and 16% utilized patients in inpatient medical settings. Of studies involving patients with a pre-existing diagnosis, 44% were specific to cardiovascular disease (CVD), 36% to cancer, 9% to renal disease, and the remaining 11% had a variety of conditions including neurological disease. Research reports most often (81%) considered all-cause mortality, but some restricted evaluations to mortality associated with cancer (9%), CVD (8%), or other causes (2%). Participants were followed for an average of 7.5 years (*SD* = 7.1, range = 3 months to 58 years), with an average of 29% of the participants dying within each study's follow-up period.

**Table 1:** Overview of the 148 studies included in the meta-analysis.

Source	Total Number of Participants	Average Age at Intake	Location of Study	Study Length	Cause of Mortality	Social Relationship Measure	Original Statistic Metric	lnOR	Standard Error
Ahern et al., 1990 [29]	353	50	USA	1 y	All-cause	Functional	M & SD	0.27	0.36
Alter et al., 2006 [30]	3,138	64	Canada	5 y 4 m	CVD	Combined	Chi	0.06	0.15
Anstey et al., 2002 [31]	2,065	78	Australia	9 y	All-cause	Structural	Freq	0.44	0.09
Astrand et al., 1989 [32]	391	50	Sweden	22 y	All-cause	Combined	OR	0.00	0.18
Avlund et al., 1998 [33]	727	70	Denmark	11 y	All-cause	Combined	OR	0.40	0.16
Avlund et al., 2004 [34]	565	75	Denmark, Finland	5 y	All-cause	Structural	OR	0.54	0.22
Barefoot et al., 2005 [35]	3,109	58	Denmark	7 y 2 m	All-cause	Structural	p	0.15	0.12
Berkman and Syme, 1979 [36]	4,765	47	USA	9 y	All-cause	Structural	Freq	0.60	0.30
Berkman et al., 2004 [37]	3,495	45	France	10 y	All-cause	Structural	RR	1.61	0.14
Birket-Smith et al., 1989 [38]	128	73	Denmark	1 y	All-cause	Structural	R	0.37	0.33
Blazer, 1982 [39]	331	72	USA	2 y 6 m	All-cause	Combined	RR	1.05	0.30
Blazer et al., 2001 [40]	3,664	73	USA	3 y	All-cause	Combined	OR	0.15	0.10
Bowling, 1989 [41]	503	73	UK	6 y	All-cause	Structural	Chi	0.51	0.16
Brown et al., 2003 [42]	846	NR	USA	5 y	All-cause	Combined	OR	0.01	0.22
Brummet et al., 2005 [43]	2,711	62	USA	11 y 1m	All-cause	Functional	p	0.25	0.17
Burg et al., 2005 [44]	1,899	75	USA	2 y 5 m	All-cause	Combined	Freq	1.39	0.28
Burns et al., 2005 [45]	147	63	Australia	7 y 4 m	Cancer	Combined	Combin	0.45	0.31
Butow et al., 1999 [46]	125	55	Australia	2 y	Cancer	Combined	p	0.35	0.33
Bygren et al., 1996 [47]	12,675	43	Sweden	9 y	All-cause	Structural	Freq	0.41	0.07
Case et al., 1992 [48]	1,195	59	Canada, USA	4 y 2 m	CVD	Structural	RR	0.68	0.25
Cassileth et al., 1988 [49]	203	60	USA	8 y	Cancer	Structural	Combin	−0.03	0.26
Ceria et al., 2001 [50]	1,786	78	USA	6 y	All-cause	Structural	RR	1.01	0.12
Chacko et al., 1996 [51]	94	53	USA	4 y 8 m	CVD	Functional	Chi	0.92	0.39
Christensen et al., 1999 [52]	133	29	USA	58 y 2m	All-cause	Combined	Chi	0.98	0.32
Christensen et al., 1994 [53]	78	54	USA	5 y	All-cause	Functional	Chi	0.98	0.44
Cohen et al., 1987 [54]	155	73	USA	3 y	All-cause	Structural	T	0.65	0.30
Colon et al., 1991 [55]	100	30	USA	2 y	Cancer	Functional	Chi	0.86	0.38
Cornman et al., 2003 [56]	4,049	NR	Taiwan	3 y	All-cause	Structural	OR	0.17	0.06
Coyne et al., 2001 [57]	189	53	USA	4 y	CVD	Functional	RR	0.99	0.26
Cree et al., 2000 [58]	558	82	Canada	4 m	All-cause	Functional	OR	0.30	0.34
Cuijpers, 2001 [59]	424	85	Netherlands	1 y	All-cause	Functional	OR	−0.10	0.31
Dalgard & Haheim, 1998 [60]	1,002	46	Norway	17 y	All-cause	Structural	p	0.23	0.15
Devins et al., 1990 [61]	97	40	Canada	4 y	Other	Structural	R	−0.025	0.38
Dickens et al., 2004 [62]	556	60	UK	1 y	CVD	Functional	p	0.65	0.45
Ell et al., 1992 [63]	294	61	USA	6 y 11m	All-cause	Combined	p	−0.15	0.21
Eng et al., 2002 [64]	16,242	55	USA	10 y	All-cause	Structural	RR	0.42	0.06
Engedal,1996 [65]	334	82	Norway	3 y	All-cause	Structural	M & SD	0.62	0.20
Farmer et al., 1996 [66]	320	60	USA	4 y 7m	All-cause	Combined	RR	0.81	0.22
Forster & Stoller, 1992 [67]	363	74	USA	7 y	All-cause	Combined	LnOR	−0.20	0.22
Frasure-Smith et al., 2000 [68]	887	59	Canada	1 y	CVD	Functional	p	0.09	0.12
Frick et al., 2005 [69]	99	55	Germany	3 y 11m	Cancer	Combined	p	0.23	0.35
Fry and Debats, 2006 [70]	380	75	Canada	5 y 11m	All-cause	Combined	RR	0.78	0.24
Fuhrer et al., 1999 [71]	3,777	76	France	5 y	All-cause	Combined	RR	0.38	0.13
Funch & Marshall, 1983 [72]	208	51	USA	20 y	Cancer	Structural	Combin	0.17	0.26
Ganzini et al., 1997 [73]	100	73	USA	2 y 6m	All-cause	Combined	Combin	0.15	0.25
Gellert et al., 1993 [74]	136	47	USA	10 y	Cancer	Functional	RR	−0.24	0.40
Giles et al., 2005 [75]	1,477	80	Australia	10 y	All-cause	Structural	p	0.21	0.10
Giraldi et al., 1997 [76]	74	51	Italy	6 y	Cancer	Functional	M & SD	0.14	0.43
Glass et al., 1999 [77]	1,380	72	USA	13 y	All-cause	Structural	RR	0.42	0.20
Goldman et al., 1995 [78]	7,478	77	USA	6 y	All-cause	Structural	OR	0.30	0.06
Goodwin et al., 1996 [79]	328	72	USA	10 y	All-cause	Structural	p	0.62	0.20
Gorkin et al.,1993 [80]	1,146	61	USA	10 m	All-cause	Functional	Freq	0.23	0.28
Grand et al., 1990 [81]	645	75	France	4 y	All-cause	Combined	OR	0.40	0.22
Greenfield et al., 2002 [82]	5,092	NR	USA	11 y	All-cause	Structural	RR	0.38	0.14
Greenwood et al., 1995 [83]	1,274	59	UK	4 y	All-cause	Structural	RR	0.43	0.17
Grodner et al., 1996 [84]	110	63	USA	6 y	All-cause	Combined	M & SD	0.50	0.35
Gustafsson et al., 1998 [85]	421	81	Sweden	6 y	All-cause	Structural	OR	0.24	0.19
Hall et al., 1993 [86]	5,921	60	Sweden	11 y	CVD	Structural	OR	0.23	0.15
Helweg-Larsen, 2003 [87]	6,617	44	Denmark	13 y	All-cause	Combined	RR	0.74	0.05
Herndon et al., 1999 [88]	206	61	USA	4 y 2 m	Cancer	Functional	p	0.16	0.26
Hill et al., 2005 [89]	3,050	78	USA	8 y	All-cause	Combined	p	0.08	0.07
Hirdes & Forbes, 1992 [90]	259	45	Canada	20 y	All-cause	Combined	RR	0.55	0.29
Ho, 1991 [91]	946	77	China	2 y	All-cause	Combined	RR	0.55	0.24
House et al., 1982 [92]	2,754	52	USA	12 y	All-cause	Structural	Combin	0.27	0.17
Hummer et al., 1999 [93]	21,204	43	USA	8 y	All-cause	Structural	Freq	0.45	0.05
Iribarren et al., 2005 [94]	5,108	25	USA	16 y	All-cause	Structural	Combin	0.60	0.21
Irvine et al., 1999 [95]	634	64	Canada	2 y	All-cause	Structural	RR	0.01	0.32
Iwasaki et al., 2002 [96]	11,560	55	Japan	7 y	All-cause	Combined	RR	0.22	0.11
Johnson et al., 2005 [97]	3,698	43	USA	5 y	All-cause	Combined	p	0.18	0.10
Johnson et al., 1996 [98]	1,257	64	Sweden	14 y	CVD	Functional	RR	0.21	0.15
Jorm et al., 1991 [99]	228	79	Australia	5 y	All-cause	Functional	M & SD	0.24	0.24
Juon et al., 2003 [100]	1,091	6	USA	28 y	All-cause	Structural	OR	0.60	0.35
Jylhä and Aro, 1989 [101]	936	NR	Finland	6 y 6 m	All-cause	Combined	p	0.32	0.12
Kaplan et al., 1988 [102]	5,320	49	Finland	5 y	All-cause	Structural	OR	0.75	0.18
Kaplan et al., 1994 [103]	2,501	53	Finland	5 y 11m	All-cause	Combined	RR	0.27	0.19
Kawachi et al., 1996 [104]	18,702	60	USA	4 y	All-cause	Structural	RR	0.50	0.17
Keller et al., 2003 [105]	654	78	USA	10 y	All-cause	Structural	p	0.53	0.14
Kiely et al., 2000 [106]	916	87	USA	4 y 6 m	All-cause	Structural	p	0.23	0.12
Kimmel et al., 2000 [107]	174	54	USA	5 y	All-cause	Functional	p	0.73	0.17
Korten et al., 1999 [108]	752	70	Australia	4 y	All-cause	Combined	Combin	0.20	0.13
Krause, 1997 [109]	2,209	68	UK	11 y	All-cause	Combined	OR	−0.03	0.10
Krause, 2006 [110]	976	74	USA	3 y	All-cause	Combined	OR	0	0.18
Kroenke et al., 2006 [111]	2,835	59	USA	12 y	All-cause	Structural	RR	0.45	0.22
La Cour et al., 2005 [112]	734	70	Denmark	20 y	All-cause	Structural	p	0.45	0.14
Lee & Rotheram-Borus, 2001 [113]	307	38	USA	2 y 4 m	Other	Functional	p	0.54	0.21
Lehto et al., 2006 [114]	101	54	Finland	9 y	Cancer	Functional	p	0.97	0.38
Lennartsson and Silverstein, 2001 [115]	463	82	Sweden	4 y	All-cause	Structural	RR	0.40	0.17
Ljungquist et al., 1995 [116]	956	70	Sweden	10 y	All-cause	Combined	OR	1.03	0.16
Lund et al., 2002 [117]	1,265	60	Denmark	8 y	All-cause	Structural	p	0.37	0.16
Lund et al., 2000 [118]	894	79	Denmark	8 y	All-cause	Structural	OR	0.30	0.21
Lyyra and Heikkinen, 2006 [119]	206	80	Finland	10 y	All-cause	Combined	Combin	0.25	0.30
Maier & Smith, 1999 [120]	513	85	Germany	6 y	All-cause	Functional	Combin	0.33	0.16
Malmstrom et al., 2001 [121]	22,236	47	Sweden	8 y	All-cause	Structural	RR	0.30	0.07
McClellan et al., 1993 [122]	210	55	USA	1 y	All-cause	Functional	M & SD	0.24	0.34
Merlo et al., 2000 [123]	491	68	Sweden	10 y	All-cause	Combined	Freq	0.63	0.19
Mertens et al., 1996 [124]	1,869	62	USA	4 y	All-cause	Structural	M & SD	0.56	0.08
Morris et al., 1993 [125]	91	60	USA	10 y	All-cause	Structural	T	0.81	0.40
Murata et al., 2005 [126]	1,994	73	Japan	7 y 4 m	All-cause	Combined	p	0.12	0.11
Murberg and Bru, 2001 [127]	119	66	Norway	2 y	CVD	Combined	p	0.27	0.34
Musick et al., 2004 [128]	3,617	47	USA	7 y 6 m	All-cause	Combined	R	0.17	0.06
Nakanishi and Tatara, 2000 [129]	1,285	74	Japan	5 y 6 m	All-cause	Structural	p	0.26	0.10
Nordentoft et al., 1993 [130]	974	41	Denmark	10 y	All-cause	Structural	p	0.42	0.12
Olsen et al., 1991 [131]	1,637	79	Denmark	15 y 6m	All-cause	Combined	p	0.14	0.11
Oman and Reed, 1998 [132]	2,023	75	USA	5 y 7 m	All-cause	Structural	P	0.20	0.11
Orrell et al., 2000 [133]	60	80	UK	3 y	All-cause	Combined	p	0.62	0.48
Orth-Gomer and Johnson, 1987 [134]	17,433	49	Sweden	6 y	?	Structural	RR	1.31	0.07
Orth-Gomer and Unden, 1990 [135]	147	57	Sweden	10 y	All-cause	Structural	T	0.86	0.40
Ostbye et al., 2006 [136]	4,012	77	USA	10 y	All-cause	Combined	OR	0.54	0.09
Oxman et al., 1995 [137]	232	76	USA	6 m	CVD	Combined	Combin	0.33	0.46
Parkerson and Gutman, 2000 [138]	103	63	USA	1 y	All-cause	Structural	OR	1.65	0.58
Pennix et al., 1997 [139]	2,829	70	Netherlands	3 y	All-cause	Combined	Freq	0.30	0.15
Rasulo et al., 2005 [140]	1,734	81	Denmark	6 y	All-cause	Structural	p	0.11	0.09
Reuben et al., 1992 [141]	259	73	USA	4 y 3 m	All-cause	Combined	R	0.52	0.22
Reynolds et al., 1994 [142]	1,011	53	USA	5 y	Cancer	Combined	p	0.19	0.17
Rodriguez-Artalejo et al., 2006 [143]	251	77	Spain	7 m	CVD	Structural	p	0.17	0.33
Rosengren et al., 1998 [144]	717	50	Sweden	12 y	All-cause	Combined	Freq	0.64	0.28
Roy et al., 1996 [145]	547	80	USA	4 y	All-cause	Structural	RR	0.76	0.15
Rozzini et al., 1991 [146]	1,201	73	Italy	3 y	All-cause	Structural	Freq	0.94	0.20
Ruberman et al., 1984 [147]	2,320	50	USA	3 y	All-cause	Structural	Chi	0.39	0.08
Rutledge et al., 2003 [148]	7,524	71	USA	6 y	All-cause	Combined	RR	0.53	0.05
Rutledge et al., 2004 [149]	503	59	USA	2 y 4 m	All-cause	Combined	M & SD	0.99	0.37
Saito-Nakaya et al., 2006 [150]	238	62	Japan	7 y 6 m	All-cause	Combined	Freq	−0.07	0.35
Schoenbach et al., 1986 [151]	791	55	USA	2 y	All-cause	Structural	Freq	0.80	0.19
Seeman et al., 1993 [152]	1,420	74	USA	5 y	All-cause	Combined	p	1.83	0.17
Shahatahmasebi et al., 1992 [153]	534	72	UK	8 y	All-cause	Combined	Chi	0.40	0.16
Shmotkin et al., 2003 [154]	1,174	84	Israel	8 y	All-cause	Structural	p	−0.09	0.12
Shye et al., 1995 [155]	455	72	USA	15 y	All-cause	Structural	Freq	0.80	0.21
Silverstein and Bengston, 1991 [156]	435	67	USA	14 y	All-cause	Combined	OR	0.03	0.16
Soler-Vila et al., 2003 [157]	322	54	USA	10 y	All-cause	Combined	M & SD	0.29	0.20
Stavraky et al., 1988 [158]	224	59	Canada	1 y	Cancer	Combined	Freq	0.55	0.35
Stek et al., 2005 [159]	476	85	Netherlands	5 y	All-cause	Functional	p	0.35	0.21
Sturdy et al., 2002 [160]	1,066	53	UK	5 y	All-cause	Structural	OR	0.17	0.35
Sugisawa et al., 1994 [161]	1,943	69	Japan	3 y	All-cause	Combined	p	0.03	0.19
Sun and Lui, 2006 [162]	7,938	92	China	2 y	All-cause	Structural	R	0.67	0.04
Temkin-Greener et al., 2004 [163]	3,138	79	USA	2 y	All-cause	Combined	p	0.21	0.10
Thomas et al., 1997 [164]	424	63	Canada, USA	3 y 11m	CVD	Functional	M & SD	0.10	0.18
Tucker et al., 1996 [165]	1,077	12	USA	41 y	All-cause	Structural	p	0.27	0.12
Vaillant et al., 1998 [166]	223	20	USA	25 y	All-cause	Combined	OR	1.15	0.37
Vogt et al., 1992 [167]	2,396	47	USA	15 y	All-cause	Structural	p	0.20	0.08
Walter-Ginzburg et al., 2002 [168]	1,340	83	Israel	8 y	All-cause	Combined	Freq	0.23	0.11
Waxler-Morrison et al., 1991 [169]	118	45	Canada	4 y	Cancer	Structural	p	0.27	0.36
Weihs et al., 2005 [170]	90	52	USA	9 y	Cancer	Structural	Combin	0.61	0.40
Welin et al., 2000 [171]	275	55	Sweden	10 y	All-cause	Combined	p	0.44	0.22
Welin et al., 1992 [172]	959	60	Sweden	12 y	All-cause	Combined	Combin	0.52	0.17
Wilkins, 2003 [173]	2,107	75	Canada	6 y	All-cause	Combined	RR	0.05	0.12
Woloshin et al., 1997 [174]	37	67	Canada	1 y	All-cause	Functional	OR	1.87	0.61
Yasuda et al., 1997 [175]	806	74	USA	5 y	All-cause	Combined	Freq	0.27	0.19
Zuckerman et al., 1984 [176]	398	72	USA	2 y	All-cause	Combined	Combin	0.09	0.18

Chi, chi-square; Combin, combined statistics; Freq, frequency counts; m, months; M & SD, means and standard deviations; NR, not reported; OR, odds ratio; RR, risk ratio; p, level of statistical significance; t, t-scores; y, years.

### Omnibus Analysis

Across 148 studies, the random effects weighted average effect size was OR = 1.50 (95% confidence interval [CI] = 1.42 to 1.59), which indicated a 50% increased likelihood of survival as a function of stronger social relations. Odds ratios ranged from 0.77 to 6.50, with substantial heterogeneity across studies (*I^2^* = 81% [95% CI = 78% to 84%]; *Q*_(147)_ = 790, *p*<0.001; τ^2^ = 0.07), suggesting that systematic effect size variability was unaccounted for. Thus factors associated with the studies themselves (e.g., publication status), participant characteristics (e.g., age, health status), and the type of evaluation of social relationships (e.g., structural social networks versus perceptions of functional social support) may have moderated the overall results. We therefore conducted additional analyses to determine the extent to which these variables moderated the overall results.

**Figure 2:**
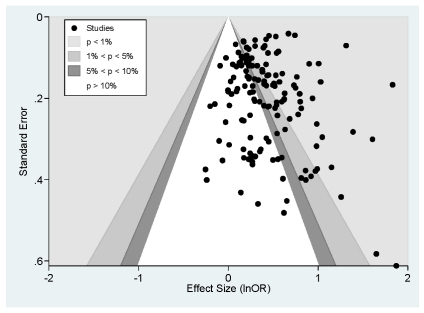
Contour enhanced funnel plot.

To assess the possibility of publication bias [177], we conducted several analyses. First, we calculated the fail-safe *N* [177] to be 4,274, which is the theoretical number of unpublished studies with effect sizes averaging zero (no effect) that would be needed to render negligible the omnibus results. Second, we employed the “trim and fill” methodology described by Duval and Tweedie [178],[179] to estimate the number of studies missing due to publication bias, but this analysis failed to reveal any studies that would need to be created on the opposite side of the distribution, meaning that adjustment to the omnibus effect size was unnecessary. Third, we calculated both Egger's regression test and the alternative to that test recommended by Peters and colleagues [180] that is better suited to data in lnOR format. The results of both analyses failed to reach statistical significance (*p*>0.05). Finally, we plotted a contour-enhanced funnel plot (Figure 2) [181]. The data obtained from this meta-analysis were fairly symmetrical with respect to their own mean; fewer than ten studies were “missing” on the left side of the distribution that would have made the plot symmetrical. Based on these several analyses, publication bias is unlikely to threaten the results.

### Moderation by Social Relationship Assessment, and by Participant and Study Characteristics

Given that structural versus functional components of social relationships may influence health in different ways [11],[12], the high degree of heterogeneity observed in the omnibus results may have been due in part to differences between the components of social relationships evaluated within and across studies. Hence the remaining analyses separately evaluate effect sizes obtained from structural, functional, and combined (structural and functional) measures of social relationships. Table 2 provides definitions of the types and subtypes of social relationships evaluated.

**Table 2:** Descriptive coding of the measures used to assess social relationships.

Type of Measure		Description	Example of Measure
***Functional***		***Functions provided or perceived to be available by social relationships***	
	Received support	Self-reported receipt of emotional, informational, tangible, or belonging support	• Inventory of Social Supportive Behaviors [213]• UCLA Social Support Interview [214],[215]• Social Support Behaviors Scale [216]
	Perceptions of social support	Perception of availability of emotional, informational, tangible, or belonging support if needed.	• EPESE support questions [217]• Malmo Social Support Scale [218]• Social Support Questionnaire [219]• Interpersonal Support Evaluation List [220]
	Perception of loneliness	Feelings of isolation, disconnectedness, and not belonging	• Loneliness Scale [221]• UCLA Loneliness Scale [222]
***Structural***		***The existence and interconnections among differing social ties and roles***	
	Marital status	married versus other	• Binary item: Married yes, no• Married, never married, divorced, separated, widowed
	Social networks	network density or size, number of social contacts	• Convoy measure [223]• Social Network List [224]
	Social integration	Participation in a broad range of social relationships; including active engagement in a variety of social activities or relationships, and a sense of communality and identification with one's social roles.	• Malmo Influence, Contact, & Anchorage Measure [225]• Social Network Index [226],[227]• Social Participation Scale [92]
	Complex measures of social integration	A single measure that assesses multiple components of social integration such as marital status, network size and network participation.	• Social Network Index [36]• Social Network Questionnaire [228]• Social Connections Index [102]• Rand Social Health Battery [229]
	Living alone	Living alone versus living with others	• Binary item: yes, no• Number of people in household
	Social isolation	Pervasive lack of social contact or communication, participation in social activities, or confidant	• Social Isolation Scale [82]
***Combined***		***Assessment of both structural and functional measures***	
	Multifaceted Measurement	Multiple measures obtained that assess more than one of the above conceptualizations.	

#### Structural aspects of social relationships

**Figure 3:**
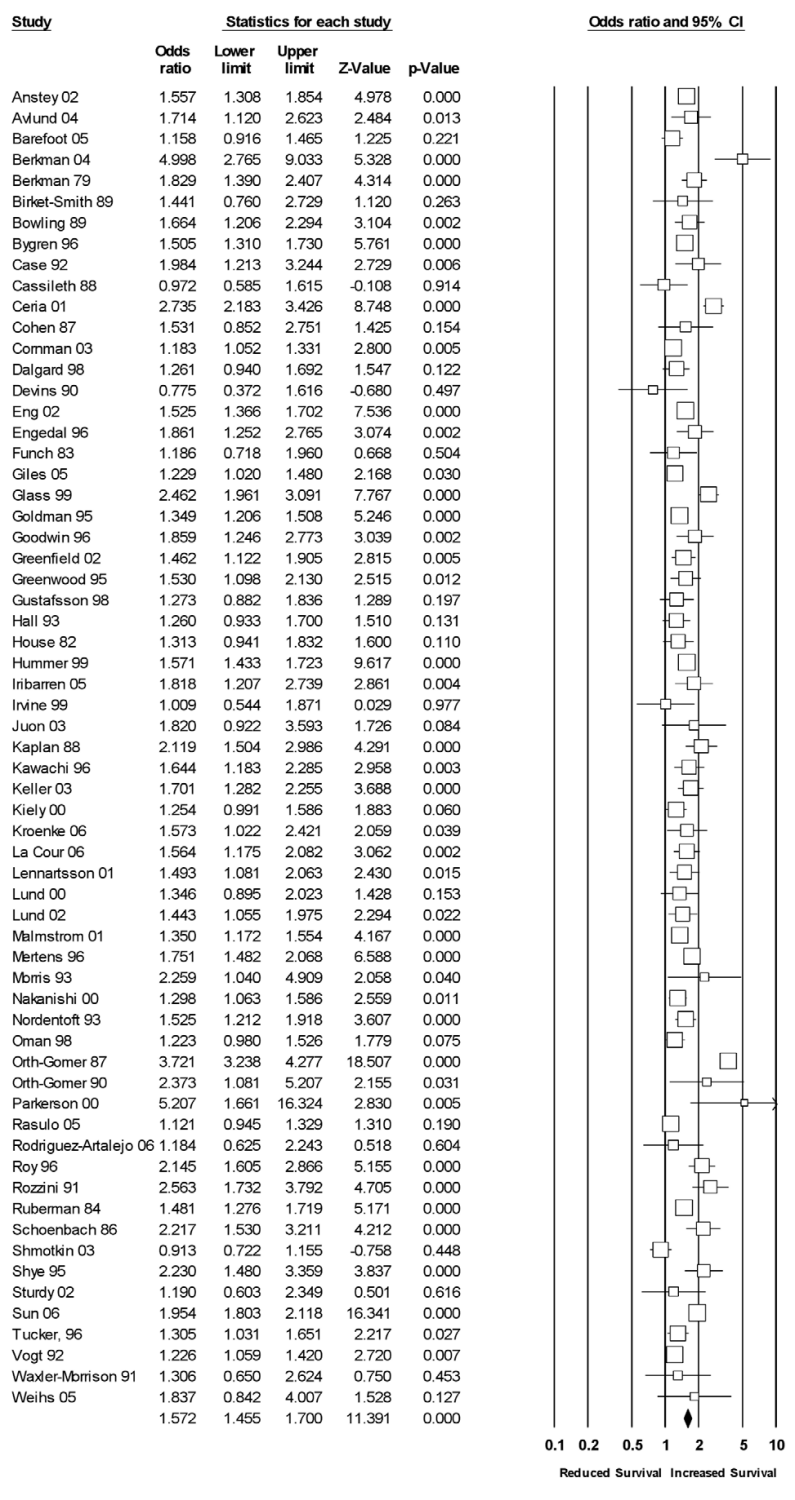
Forest plot of structural measures.

Sixty-three studies had data exclusive to structural measures of social relationships (see Figure 3). Across these studies, the random effects weighted average effect size was OR = 1.57 (95% CI = 1.46 to 1.70), which value fell within the CI of the omnibus results reported previously. The heterogeneity across studies was still quite large (*I^2^* = 84% [95% CI = 80% to 87%]; *Q*_(62)_ = 390, *p*<0.001; τ^2^ = 0.07), so we undertook metaregression with prespecified participant and study characteristics.

Metaregression is an analogue to multiple regression analysis for effect sizes. Its primary purpose is to ascertain which continuous and categorical (dummy coded) variables predict variation in effect size estimates. Using random effects weighted metaregression, we examined the simultaneous association (with all variables entered into the model) between effect sizes and prespecified participant and study characteristics (Table 3). To examine the most precise effect size estimates available and to increase the statistical power associated with this analysis, we shifted the unit of analysis [24] and extracted effect sizes within studies that were specific to measures of structural aspects of social relationships. That is, if a study contained effect sizes from both structural and functional types of social relationships, we extracted the structural types for this analysis (with identical subtypes aggregated), which resulted in a total of 230 unique effect sizes across 116 studies. A total of 18% of the variance in these effect sizes was explained in the metaregression (*p*<0.001). As can be seen in Table 3, effect sizes based on data controlling for other variables were lower in magnitude than those based on raw data. Moreover, effect sizes differed in magnitude across the subtype of structural social relationships measured. Complex measures of social integration were associated with larger effect size values than measures of social participation. Binary measures of whether participants lived alone (yes/no) were associated with smaller effect size values. Average random effects weighted odds ratios for the various subtypes of social relationships are reported in Table 4.

**Table 3:** Random effects metaregression for effect size estimates of structural social relationships.

Variable	B	SE	*p*	β
(Constant)	0.535	0.238	0.02	0.00
Participants' average age^a^	−0.002	0.002	0.49	−0.06
Participant sex composition^b^				
100% Female	0.038	0.066	0.57	0.04
100% Male	0.049	0.068	0.48	0.05
Participant initial health^c^	−0.103	0.085	0.23	−0.10
Cause of mortality^d^				
Cardiovascular disease	0.081	0.161	0.61	0.03
Cancer	−0.208	0.139	0.13	−0.12
Length of follow-up evaluation (y)	−0.003	0.005	0.54	−0.05
Measure of social relationships^e^				
Living alone	−0.265	0.106	0.013	−0.18
Marital status	−0.097	0.074	0.19	−0.10
Social isolation	−0.144	0.178	0.42	−0.05
Social networks	−0.050	0.071	0.48	−0.06
Complex measures of integration	0.255	0.095	0.007	0.20
Geographic region of study^f^				
Asia	0.057	0.154	0.71	0.05
Europe	0.221	0.134	0.10	0.25
North America	0.057	0.134	0.69	0.07
Statistically controlled estimate^g^	−0.147	0.058	0.01	−0.17

a Age at study initiation.

b Contrasted with reports in which males and females were combined.

c Individuals with a pre-existing medical condition contrasted with community samples.

d Contrasted with all cause and all other causes.

e Contrasted with measures of social participation; see Table 2 for descriptions of each kind of measure.

f Contrasted with all other world regions combined.

g Contrasted with estimates based on raw data.

β, standardized beta; B, unstandardized beta; SE, standard error.

**Table 4:** Weighted average effect sizes across different measures of social relationships.

Type of Measure		k	*OR*	95% CI
*Functional*	Received social support	9	1.22	[0.91, 1.63]
	Perceptions of social support	73	1.35	[1.22, 1.49]
	Loneliness (inversed)	8	1.45	[1.08, 1.94]
*Structural*	Living alone (inversed)	17	1.19	[0.99, 1.44]
	Marital status (married versus other)	62	1.33	[1.20, 1.48]
	Social isolation (inversed)	8	1.40	[1.06, 1.86]
	Social networks	71	1.45	[1.32, 1.59]
	Social integration	45	1.52	[1.36, 1.69]
	Complex measures of social integration	30	1.91	[1.63, 2.23]
*Combined structural and functional*	Multifaceted measurement	67	1.47	[1.34, 1.60]

These analyses shifted the units of analysis, with distinct effect size estimates within studies used within different categories of measurement, such that many studies contributed more than one effect size but not more than one per category of measurement.

*OR*, odds ratio, transformed from random effects weighted lnOR.

#### Functional aspects of social relationships

**Figure 4:**
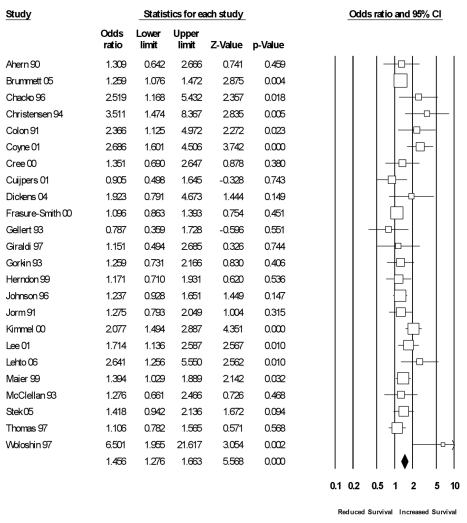
Forest plot of functional measures.

Twenty-four studies had data exclusive to functional measures of social relationships (see Figure 4). Across these studies, the random effects weighted average effect size was OR = 1.46 (95% CI = 1.28 to 1.66), which value fell within the CI of the omnibus results reported previously. There was moderate heterogeneity across studies (*I^2^* = 47% [95% CI = 16% to 68%]; *Q*_(23)_ = 44, *p*<0.01; τ^2^ = 0.04), so we conducted a random effects metaregression using the same variables and analytic procedures described previously. We extracted 87 unique effect sizes that were specific to measures of functional social relationships within 72 studies. A total of 16.5% of the variance in these effect sizes was explained in the metaregression, but the model did not reach statistical significance (*p* = 0.46). The results were not moderated by any of the specified participant characteristics (age, sex, initial health status, cause of mortality) or study characteristics (length of follow-up, geographic region, statistical controls).

#### Combined assessments of social relationships

**Figure 5:**
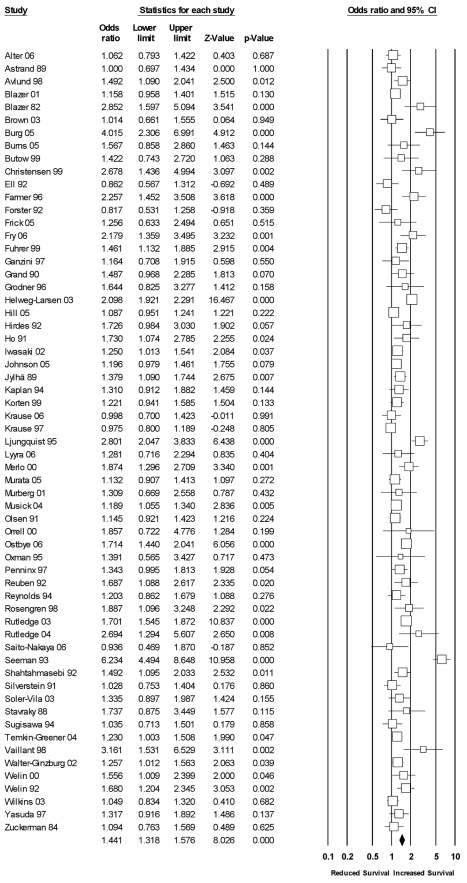
Forest plot of combined measures.

Sixty-one studies had combined data of both structural and functional measures of social relationships (see Figure 5). Across these studies, the random effects weighted average effect size was OR = 1.44 (95% CI = 1.32 to 1.58). A large degree of heterogeneity characterized studies (*I^2^* = 82% [95% CI = 78% to 86%]; *Q*_(60)_ = 337, *p*<0.001; τ^2^ = 0.09), and we conducted a random effects metaregression using the same variables and analytic procedures described previously. We extracted 64 unique effect sizes that evaluated combined structural and functional measures of social relationships within 61 studies. The metaregression explained only 6.8% of the variance in these effect sizes, and the model failed to reach statistical significance (*p* = 0.95). None of the variables in the metaregression moderated the results.

## Discussion

**Figure 6:**
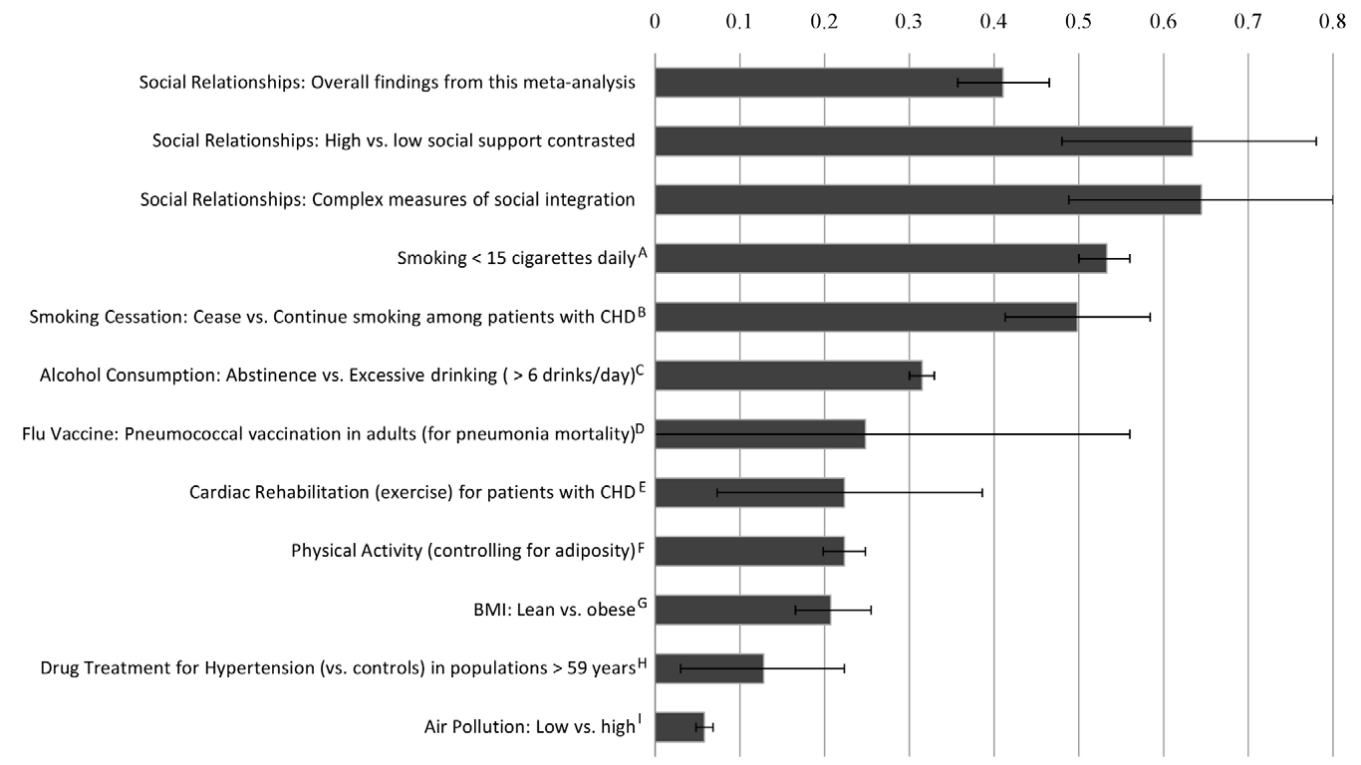
Comparison of odds (lnOR) of decreased mortality across several conditions associated with mortality. Note: Effect size of zero indicates no effect. The effect sizes were estimated from meta analyses: ; A = Shavelle, Paculdo, Strauss, and Kush, 2008 [205]; B = Critchley and Capewell, 2003 [206]; C = Holman, English, Milne, and Winter, 1996 [207]; D = Fine, Smith, Carson, Meffe, Sankey, Weissfeld, Detsky, and Kapoor, 1994 [208]; E = Taylor, Brown, Ebrahim, Jollife, Noorani, Rees et al., 2004 [209]; F, G = Katzmarzyk, Janssen, and Ardern, 2003 [210]; H = Insua, Sacks, Lau, Lau, Reitman, Pagano, and Chalmers, 1994 [211]; I = Schwartz, 1994 [212].

Cumulative empirical evidence across 148 independent studies indicates that individuals' experiences within social relationships significantly predict mortality. The overall effect size corresponds with a 50% increase in odds of survival as a function of social relationships. Multidimensional assessments of social integration yielded an even stronger association: a 91% increase in odds of survival. Thus, the magnitude of these findings may be considered quite large, rivaling that of well-established risk factors (Figure 6). Results also remained consistent across a number of factors, including age, sex, initial health status, follow-up period, and cause of death, suggesting that the association between social relationships and mortality may be generalized.

The magnitude of risk reduction varied depending on the type of measurement of social relationships (see Table 4). Social relationships were most highly predictive of reduced risk of mortality in studies that included multidimensional assessments of social integration. Because these studies included more than one type of social relationship measurement (e.g., network based inventories, marital status, etc.), such a measurement approach may better represent the multiple pathways (described earlier) by which social relationships influence health and mortality [182]. Conversely, binary evaluations of living alone (yes/no) were the least predictive of mortality status. The reliability and validity of measurement likely explains this finding, and researchers are encouraged to use psychometrically sound measures of social relationships (e.g., Table 2). For instance, while researchers may be tempted to use a simple single-item such as “living alone” as a proxy for social isolation, it is possible for one to live alone but have a large supportive social network and thus not adequately capture social isolation. We also found that social isolation had a similar influence on likelihood of mortality compared with other measures of social relationships. This evidence qualifies the notion of a threshold effect (lack of social relationships is the only detrimental condition); rather, the association appears robust across a variety of types of measures of social relationships.

This meta-analysis also provides evidence to support the directional influence of social relationships on mortality. Most of the studies (60%) involved community cohorts, most of whom would not be experiencing life-threatening conditions at the point of initial evaluation. Moreover, initial health status did not moderate the effect of social relationships on mortality. Although illness may result in poorer or more restricted social relationships (social isolation resulting from physical confinement), such that individuals closer to death may have decreased social support compared to healthy individuals, the findings from these studies indicate that general community samples with strong social relationships are likely to remain alive longer than similar individuals with poor social relations. However, causality is not easily established. One cannot randomly assign human participants to be socially isolated, married, or in a poor-quality relationship. A similar dilemma characterizes virtually all lifestyle risk factors for mortality: for instance, one cannot randomly assign individuals to be smokers or nonsmokers. Despite such challenges, “smoking represents the most extensively documented cause of disease ever investigated in the history of biomedical research” [183]. The link between social relationships and mortality is currently much less understood than other risk factors; nonetheless there is substantial experimental, cross-sectional, and prospective evidence linking social relationships with multiple pathways associated with mortality (see [182] for review). Existing models for reducing risk of mortality may be substantially strengthened by including social relationship factors.

Notably, the overall effect for social relationships on mortality reported here may be a conservative estimate. Many studies included in the meta-analysis utilized single item measures of social relations, yet the magnitude of the association was greatest among those studies utilizing complex assessments. Moreover, because many studies statistically adjusted for standard risk factors, the effect may be underestimated, since some of the impact of social relationships on mortality may be mediated through such factors (e.g., behavior, diet, exercise). Additionally, most measures of social relations did not take into account the *quality* of the social relationships, thereby assuming that all relationships are positive. However, research suggests this is not the case, with negative social relationships linked to greater risk of mortality [184],[185]. For instance, marital status is widely used as a measure of social integration; however, a growing literature documents its divergent effects based on level of marital quality [186],[187]. Thus the effect of positive social relationships on risk of mortality may actually be much larger than reported in this meta-analysis, given the failure to account for negative or detrimental social relationships within the measures utilized across studies.

Other possible limitations of this review should be acknowledged. Statistical controls (e.g., age, sex, physical condition, etc.) employed by many of the studies rule out a number of potentially confounding variables that might account for the association between social relationships and mortality. However, studies used an inconsistent variety of controlling variables, and some reports involved raw data (Table 1). Although effect size magnitude was diminished by the inclusion of statistical controls only within the data obtained by measures of structural social relationships (but not functional or combined measures), future research can better specify which variables are most likely to impact the overall association. It must also be acknowledged that existing data primarily represent research conducted in North America and Western Europe. Although we found no differences across world region, future reviews inclusive of research written in all languages (not only English) with participants better representing other world regions may yield better estimates across populations.

Approximately two decades after the review by House and colleagues [1], a generation of empirical research validates their initial premise: Social relationships exert an independent influence on risk for mortality comparable with well established risk factors for mortality (Figure 6). Although limited by the state of current investigations and possible omission of pertinent reports, this meta-analysis provides empirical evidence (nearly 30 times the number of studies previously reported) to support the criteria for considering insufficient social relationships a risk factor of mortality (i.e., strength and consistency of association across a wide range of studies, temporal ordering, and gradient of response) [188]. The magnitude of the association between social relationships and mortality has now been established, and this meta-analysis provides much-needed clarification regarding the social relationship factor(s) most predictive of mortality. Future research can shift to more nuanced questions aimed at (a) understanding the causal pathways by which social participation promotes health, (b) refining conceptual models, and (c) developing effective intervention and prevention models that explicitly account for social relations.

Some steps have already been taken identifying the psychological, behavioral, and physiological pathways linking social relationships to health [5],[182],[189]. Social relationships are linked to better health practices and to psychological processes, such as stress and depression, that influence health outcomes in their own right [190]; however, the influence of social relationships on health cannot be completely explained by these processes, as social relationships exert an independent effect. Reviews of such findings suggest that there are multiple biologic pathways involved (physiologic regulatory mechanisms, themselves intertwined) that in turn influence a number of disease endpoints [182],[191]–[193]. For instance, a number of studies indicate that social support is linked to better immune functioning [194]–[197] and to immune-mediated inflammatory processes [198]. Thus interdisciplinary work and perspective will be important in future studies given the complexity of the phenomenon.

Perhaps the most important challenge posed by these findings is how to effectively utilize social relationships to reduce mortality risk. Preliminary investigations have demonstrated some risk reduction through formalized social interventions [199]. While the evidence is mixed [2],[6], it should be noted that most social support interventions evaluated in the literature thus far are based on support provided from strangers; in contrast, evidence provided in this meta-analysis is based almost entirely on naturally occurring social relationships. Moreover, our analyses suggest that received support is less predictive of mortality than social integration (Table 4). Therefore, facilitating patient use of naturally occurring social relations and community-based interventions may be more successful than providing social support through hired personnel, except in cases in which patient social relations appear to be detrimental or absent. Multifaceted community-based interventions may have a number of advantages because such interventions are socially grounded and include a broad cross-section of the public. Public policy initiatives need not be limited to those deemed “high risk” or those who have already developed a health condition but could potentially include low- and moderate-risk individuals earlier in the risk trajectory [200]. Overall, given the significant increase in rate of survival (not to mention quality of life factors), the results of this meta-analysis are sufficiently compelling to promote further research aimed at designing and evaluating interventions that explicitly account for social relationship factors across levels of health care (prevention, evaluation, treatment compliance, rehabilitation, etc.).

### Conclusion

Data across 308,849 individuals, followed for an average of 7.5 years, indicate that individuals with adequate social relationships have a 50% greater likelihood of survival compared to those with poor or insufficient social relationships. The magnitude of this effect is comparable with quitting smoking and it exceeds many well-known risk factors for mortality (e.g., obesity, physical inactivity). These findings also reveal significant variability in the predictive utility of social relationship variables, with multidimensional assessments of social integration being optimal when assessing an individual's risk for mortality and evidence that social isolation has a similar influence on mortality to other measures of social relationships. The overall effect remained consistent across a number of factors, including age, sex, initial health status, follow-up period, and cause of death, suggesting that the association between social relationships and mortality may be general, and efforts to reduce risk should not be isolated to subgroups such as the elderly.

To draw a parallel, many decades ago high mortality rates were observed among infants in custodial care (i.e., orphanages), even when controlling for pre-existing health conditions and medical treatment [201]–[204]. Lack of human contact predicted mortality. The medical profession was stunned to learn that infants would die without social interaction. This single finding, so simplistic in hindsight, was responsible for changes in practice and policy that markedly decreased mortality rates in custodial care settings. Contemporary medicine could similarly benefit from acknowledging the data: Social relationships influence the health outcomes of adults.

Physicians, health professionals, educators, and the public media take risk factors such as smoking, diet, and exercise seriously; the data presented here make a compelling case for social relationship factors to be added to that list. With such recognition, medical evaluations and screenings could routinely include variables of social well-being; medical care could recommend if not outright promote enhanced social connections; hospitals and clinics could involve patient support networks in implementing and monitoring treatment regimens and compliance, etc. Health care policies and public health initiatives could likewise benefit from explicitly accounting for social factors in efforts aimed at reducing mortality risk. Individuals do not exist in isolation; social factors influence individuals' health though cognitive, affective, and behavioral pathways. Efforts to reduce mortality via social relationship factors will require innovation, yet innovation already characterizes many medical interventions that extend life at the expense of quality of life. Social relationship–based interventions represent a major opportunity to enhance not only the quality of life but also survival.

## Supporting Information

Alternative Language Abstract S1 Abstract translated into Japanese by Hideko Cannell.
(0.02 MB DOC)

Alternative Language Abstract S2 Abstract translated into Spanish by Rod Veas.
(0.03 MB DOC)

Text S1 PRISMA checklist.
(0.06 MB DOC)

Text S2 Review protocol.
(0.05 MB DOC)

## Abbreviations

CI: confidence interval
CVD: cardiovascular disease
OR: odds ratio

## References

[1] House JS, Landis KR, Umberson D (1988). Social relationships and health.. Science.

[2] Berkman LF, Blumenthal J, Burg M (2003). Effects of treating depression and low perceived social support on clinical events after myocardial infarction: the Enhancing Recovery in Coronary Heart Disease Patients (ENRICHD) Randomized Trial.. JAMA.

[3] McPherson M, Smith-Lovin L (2006). Social Isolation in America: Changes in core discussion networks over two decades.. Am Sociol Rev.

[4] Putnam RD (2000). Bowling Alone: The collapse and revival of American community.

[5] Cohen S, Gottlieb BH, Underwood LG, Cohen S, Underwood LG, Gottlieb BH (2000). Social Relationships and Health.. Measuring and intervening in social support.

[6] Cohen S, Gottlieb BH, Underwood LG (2001). Social relationships and health: challenges for measurement and intervention.. Adv Mind Body Med.

[7] Cohen S (2004). Social relationships and health.. Am Psychol.

[8] Thoits PA (1983). Multiple identities and psychological well-being: A reformulation and test of the social isolation hypothesis.. Am Sociol Rev.

[9] Brissette I, Cohen S, Seeman TE, Cohen S, Underwood LG, Gottlieb BH (2000). Measuring social integration and social networks.. Social support measurement and intervention: A guide for health and social scientists.

[10] Reinhardt JP, Boerner K, Horowitz A (2006). Good to have but not to use: Differential impact of perceived and received support on well-being.. J Soc Pers Relat.

[11] Lakey B, Cohen S, Cohen S, Underwood LG, Gottlieb BH (2000). Social support theory and measurement.. Social support measurement and intervention: A guide for health and social scientists.

[12] Cohen S, Gottlieb BH, Underwood LG, Cohen S, Underwood LG, Gottlieb BH (2000). Social relationships and health.. Social support measurement and intervention: A guide for health and social scientists.

[13] DiMatteo MR (2004). Social support and patient adherence to medical treatment: a meta-analysis.. Health Psychol.

[14] Murphy BM, Elliott PC, Le Grande MR, Higgins RO, Ernest CS (2008). Living alone predicts 30-day hospital readmission after coronary artery bypass graft surgery.. Eur J Cardiovasc Prev Rehabil.

[15] Lett HS, Blumenthal JA, Babyak MA, Catellier DJ, Carney RM (2007). Social support and prognosis in patients at increased psychosocial risk recovering from myocardial infarction.. Health Psychol.

[16] Knox SS, Adelman A, Ellison RC, Arnett DK, Siegmund K (2000). Hostility, social support, and carotid artery atherosclerosis in the National Heart, Lung, and Blood Institute Family Heart Study.. Am J Cardiol.

[17] Kop WJ, Berman DS, Gransar H, Wong ND, Miranda-Peats R (2005). Social network and coronary artery calcification in asymptomatic individuals.. Psychosom Med.

[18] Brummett BH, Barefoot JC, Siegler IC, Clapp-Channing NE, Lytle BL (2001). Characteristics of socially isolated patients with coronary artery disease who are at elevated risk for mortality.. Psychosom Med.

[19] Wang HX, Mittleman MA, Leineweber C, Orth-Gomer K (2006). Depressive symptoms, social isolation, and progression of coronary artery atherosclerosis: the Stockholm Female Coronary Angiography Study.. Psychother Psychosom.

[20] Wang HX, Mittleman MA, Orth-Gomer K (2005). Influence of social support on progression of coronary artery disease in women.. Soc Sci Med.

[21] Angerer P, Siebert U, Kothny W, Muhlbauer D, Mudra H (2000). Impact of social support, cynical hostility and anger expression on progression of coronary atherosclerosis.. J Am Coll Cardiol.

[22] Knox SS, Uvnas-Moberg K (1998). Social isolation and cardiovascular disease: an atherosclerotic pathway?. Psychoneuroendocrinology.

[23] Cohen S, Wills TA (1985). Stress, social support, and the buffering hypothesis.. Psychol Bull.

[24] Borenstein M, Hedges L, Higgins J, Rothstein H (2005).

[25] Cooper H (1998). Synthesizing research: A guide for literature reviews 3rd ed.

[26] Shrout PE, Fleiss JL (1979). Intraclass correlations: Uses in assessing rater reliability.. Psychol Bull.

[27] Hedges LV, Vevea JL (1998). Fixed- and random-effects models in meta-analysis.. Psychological Methods.

[28] Mosteller F, Colditz GA (1996). Understanding research synthesis (meta-analysis).. Annual Review of Public Health.

[29] Ahern D, Gorkin L, Anderson J, Tierney C, Hallstrom A (1990). Biobehavioral variables and mortality or cardiac arrest in the cardiac arrhythmia pilot study (CAPS).. Am J Cardiol.

[30] Alter DA, Chong A, Austin PC, Mustard C, Iron K (2006). Socioeconomic status and mortality after acute myocardial infarction.. Ann Intern Med.

[31] Anstey JK, Luszcz MA (2002). Mortality risk varies according to gender and change in depressive status in very old adults.. Psychosom Med.

[32] Astrand NE, Hanson BS, Isacsson SO (1989). Job demands, job decision latitude, job support, and social network factors as predictors of mortality in a Swedish pulp and paper company.. Br J Ind Med.

[33] Avlund K, Damsgaard MT, Holstein BE (1998). Social relations and mortality. An eleven year follow-up studey of 70 year-old men and women in Denmark.. Soc Sci Med.

[34] Avlund K, Lund R, Holstein BE, Due P, Sakari-Rantala R (2004). The impact of structural and functional characteristics of social relations as determinants of functional decline.. J Gerontol.

[35] Barefoot JC, Grobaek M, Jensen G, Schnohr, Prescott E (2005). Social network diversity and risks of ischemic heart disease and total mortality: Findings from the Copenhagen City Heart Study.. Ame J Epidemiol.

[36] Berkman LF, Syme SL (1979). Social networks, host resistance, and mortality: A nine-year follow-up study of Alameda County residents.. Am J Epidemiol.

[37] Berkman LF, Melchior M, Chastang JF, Niedhammer I, Leclerc A (2004). Social integration and mortality: A prospective study of French employees of electricity of France-Gas of France: the GAZEL Cohort.. Ame J Epidemiol.

[38] Birket-Smith M, Knudsen HC, Nissen J, Blegvad N, Køhler O (1989). Life events and social support in prediction of stroke outcome.. Psychother Psychosom.

[39] Blazer DG (1982). Social support and mortality in an elderly community population.. Am J Epidemiol.

[40] Blazer D, Hybels C, Pieper C (2001). The association of depression and mortality in elderly persons: a case for multiple, independent pathways.. J Gerontol: Medical Sciences,.

[41] Bowling A (1989). Who dies after widow(er)hood? A discriminant analysis.. Omega.

[42] Brown SL, Nesse RM, Vinokur AD, Smith DM (2003). Providing social support may be more beneficial than receiving it: Results from a prospective study of mortality.. Psychol Sci.

[43] Brummett BH, Mark DB, Siegler IC, Williams RB, Babyak MA (2005). Perceived social support as a predictor of mortality of coronary patients: Effects of smoking, sedentary behavior, and depressive symptoms.. Psychosom Med.

[44] Burg MM, Barefoot J, Berkman L, Catellier DJ, Czajkowski S (2005). ENRICHD Investigators. Low perceived social support and post-myocardial infarction prognosis in the enhancing recovery in coronary heart disease clinical trial: The effects of treatment.. Psychosom Med.

[45] Burns CM, Craft PS, Roder DM (2005). Does emotional support influence survival? Findings from a longitudinal study of patients with advanced cancer.. Support Care Cancer.

[46] Butow PN, Coates AS, Dunn SM (1999). Psychosocial predictors of survival in Metastatic Melanoma.. J Clin Oncol.

[47] Bygren LO, Konlaan BB, Johansson S (1996). Attendance at cultural events, reading books or periodicals, and making music or singing in a choir as determinants for survival: Swedish interview survey of living conditions.. BMJ.

[48] Case RB, Moss AJ, Case N, McDermott M, Eberly S (1992). Living alone after myocardial infarction.. JAMA.

[49] Cassileth BR, Walsh WP, Lusk EJ (1988). Psychosocial correlates of cancer survival: A subsequent report 3 to 8 years after cancer diagnosis.. J Clin Oncol.

[50] Ceria CD, Masaki KH, Rodriguez BL, Chen R, Yano K (2001). The relationship of psychosocial factors to total mortality among older Japanese-American men: The Honolulu Heart Program.. J Am Geriatr Soc.

[51] Chacko RC, Harper RG, Gotto J (1996). Young J. Psychiatric interview and psychometric predictors of cardiac transplant survival.. Am J Psychiatry.

[52] Christensen AJ, Dornink R, Ehlers SL, Schultz SK (1999). Social Environment and Longevity in Schizophrenia.. Psychosom Med.

[53] Christensen AJ, Wiebe JS, Smith TW, Turner CW (1994). Predictors of survival among hemodialysis patients: Effect of perceived family support.. Health Psychol.

[54] Cohen CI, Teresi J, Holmes D (1987). Social networks and mortality in an inner-city elderly population.. Int J Aging Hum Dev.

[55] Colon EA, Callies AL, Popkin MK, McGlave PB (1991). Depressed mood and other variables related to bone marrow transplantation survival in acute leukemia.. Psychosomatics.

[56] Cornman JC, Goldman N, Glei DA, Weinstein M, Chang M (2003). Social ties and perceived support: Two dimensions of social relationships and health among the elderly in Taiwan.. J Aging Health.

[57] Coyne JC, Rohrbaugh MJ, Shoham V, Sonnega JS, Nichlas JM (2001). Prognostic importance of marital quality for survival of congestive heart failure.. Am J Cardiol.

[58] Cree M, Sosklone CL, Belseck E, Hornig J, McElhaney JE (2000). Mortality and institutionalization following hip fracture.. J Am Geriatr Soc.

[59] Cuijpers P (2000). Mortality and depressive symptoms in inhabitants of residential homes.. Int J Geriatr Psychiatry.

[60] Dalgard OS, Haheim LL (1998). Psychosocial risk factors and mortality: A prospective study with special focus on social support, social participation, and locus of control in Norway.. J Epidemiol Community Health.

[61] Devins GM, Mann J, Mandin H, Paul LC, Hons RB (1990). Psychological predictors of survival in end-stage renal disease.. J Nerv Ment Dis.

[62] Dickens CM, McGowan L, Pervical C, Douglas J, Tomenson B (2004). Lack of close confidant, but not depression, predicts further cardiac events after myocardial infarction.. Heart.

[63] Ell K, Nishimoto R, Mediansky L, Mantell J, Hamovitch M (1992). Social relations, social support, and survival among patients with cancer.. J Psychosom Res.

[64] Eng PM, Rimm EB, Fitzmaurice G, Kawachi I (2002). Social ties and change in social ties in relation to subsequent total and cause-specific mortality and coronary heart disease incidence in men.. Ame J Epidemiol.

[65] Engedal K (1996). Mortality in the elderly–A 3-year follow-up of an elderly community sample.. Int J Geriatr Psychiatry.

[66] Farmer IP, Meyer PS, Ramsey DJ, Goff DC, Wear ML (1996). Higher levels of social support predict greater survival following acute myocardial infarction: The Corpus Christi heart project.. Behav Med.

[67] Forster LE, Stoller EP (1992). The impact of social support on mortality: A seven-year follow-up of older men and women.. J App Gerontol.

[68] Frasure-Smith N, Lesperance F, Gravel G, Masson A, Juneau M (2000). Social support, depression, and mortality during the first year after myocardial infarction.. Circulation.

[69] Frick E, Motzke C, Fischer N, Busch R, Bumeder I (2005). Is perceived social support a predictor of survival from patients undergoing autologous peripheral blood stem cell transplantation?. Psychooncology.

[70] Fry PS, Debats DL (2006). Sources of life strengths as predictors of late-life mortality and survivorship.. Int J Aging Hum Dev.

[71] Fuhrer R, Dufouil C, Antonucci TC, Shipley JM, Heimer C (1999). Psychological disorder and mortality in French older adults: do social relations modify the association?. Am J Epidemiol.

[72] Funch DP, Marshall J (1983). The role of stress, social support and age in survival from breast cancer.. J Psychosom Res.

[73] Ganzini L, Smith DM, Fenn DS, Lee MA (1997). Depression and mortality in medically ill older adults.. J Am Geriatr Soc.

[74] Gellert GA, Maxwell RM, Siegel BS (1993). Survival of breast cancer patients receiving adjunctive psychosocial support therapy: A 10-year follow-up study.. J Clin Oncol.

[75] Giles LC, Glonek GFV, Luszcz MA, Andrews GR (2004). Effects of social networks on 10 year survival in very old Australians: The Australian longitudinal study of aging.. J Epidemiol Community Health.

[76] Giraldi T, Rodani MG, Cartel G, Grassi L (1997). Psychosocial factors and breast cancer: A 6-year Italian follow-up study.. Psychother Psychosom.

[77] Glass TA, Mendes de Leon C, Marottoli RA, Berkman LF (1999). Population based study of social and productive activities as predictors of survival among elderly Americans.. BM J.

[78] Goldman N, Korenman S, Weinstein R (1995). Marital status and health among the elderly.. Soc Sci Med.

[79] Goodwin JS, Samet JM, Hunt WC (1996). Determinants of Survival in Older Cancer Patients.. J Natl Cancer Inst.

[80] Gorkin L, Schron EB, Brooks MM, Wiklund I, Kellen J (1993). Psychosocial predictors of mortality in the cardiac arrhythmia suppression trial-1 (CAST-1).. Am J Cardiol.

[81] Grand A, Grosclaude P, Bocquet H, Pous J, Albarede JL (1990). Disability, psychosocial factors, and mortality among the elderly in a rural French population.. J Clin Epidemiol.

[82] Greenfield TK, Rehm J, Rogers JD (2002). Effects of depression and social integration on the relationship between alcohol consumption and all-cause mortality.. Addiction.

[83] Greenwood D, Packham C, Muir K, Madeley R (1995). How do economic status and social support influence survival after initial recovery from acute myocardial infarction?. Soc Sci Med.

[84] Grodner S, Prewitt LM, Jaworski BA, Myers R, Kaplan R (1996). The impact of social support in pulmonary rehabilitation of patients with chronic obstructive pulmonary disease.. Ann Behav Med.

[85] Gustafsson TM, Isacson DGL, Thorslund M (1998). Mortality in elderly men and women in a Swedish municipality.. Age Ageing.

[86] Hall EM, Johnson JV, Tsou TS (1993). Women, occupation, and risk of cardiovascular morbidity and mortality.. Occup Med.

[87] Helweg-Larsen M, Kjoller M, Thonig H (2003). Do age and social relations moderate the relationship between self-rated health and mortality among adult Danes.. Soc Sci Med.

[88] Herndon JE, Fleishman S, Kornblith AB, Kosty M, Green MR (1999). Is quality of life predictive of the survival of patients with advanced nonsmall cell lung carcinoma.. Cancer.

[89] Hill TD, Angel JL, Ellison CG, Angel RJ (2005). Religious attendance and mortality: An 8-year follow-up of older Mexican Americans.. J Gerontol.

[90] Hirdes JP, Forbes WF (1992). The importance of social relationships, socioeconomic status, and health practices with respect to mortality among healthy Ontario males.. J Clin Epidemiol.

[91] Ho SC (1991). Health and social predictors of mortality in an elderly Chinese cohort.. Ame J Epidemiol.

[92] House JS, Robbins C, Metzner HL (1982). The association of social relationships and activities with mortality: Prospective evidence from the Tecumseh community health study.. Ame J Epidemiol.

[93] Hummer RA, Rogers RG, Nam CB, Ellison CG (1999). Religious involvement and U.S. adult mortality.. Demography.

[94] Iribarren C, Jacobs DR, Kiefe CI, Lewis CE, Matthews KA (2005). Causes and demographic, medical, lifestyle and psychosocial predictors of premature mortality: The CARDIA study.. Soc Sci Med.

[95] Irvine J, Basinski A, Baker B, Jandciu S, Paquette M (1999). Depression and risk of sudden cardiac death after acute myocardial infarction: testing for the confounding effects of fatigue.. Psychosom Med.

[96] Iwasaki M, Otani T, Sunaga R, Miyazaki H, Xiao L (2002). Social networks and mortality base on the Komo-ise cohort study in Japan.. Int J Epidemiol.

[97] Johnson JE, Finney JW, Moos RH (2005). Predictors of 5-year mortality following inpatients/residential group treatment of substance use disorders.. Addict Behav.

[98] Johnson JV, Stewart W, Hall EM, Fredlund P, Theorell T (1996). Long-term psychosocial work environment and cardiovascular mortality among Swedish men.. Am J Public Health.

[99] Jorm AF, Henderson AS, Kay DWK, Jacomb PA (1991). Mortality in relation to dementia, depression, and social integration in an elderly community sample.. Int J Geriatr Psychiatry.

[100] Juon H, Ensminger ME, Feehan M (1989). Childhood adversity and later mortality in an urban African American cohort.. Am J Public Health.

[101] Jylhä M, Aro S (1989). Social ties and survival among the elderly in Tampere, Finland.. Int J Epidemiol.

[102] Kaplan GA, Salonen JT, Cohen RD, Brand RJ, Syme SL (1988). Social connections and mortality from all causes and from cardiovascular disease: Prospective evidence from eastern Finland.. Ame J Epidemiol.

[103] Kaplan GA, Wilson TW, Cohen RD, Kauhanen J, Wu M (1994). Social functioning and overall mortality: Prospective evidence from the Kuipio ischemic heart disease risk factor study.. Epidemiology.

[104] Kawachi I, Colditz GA, Ascherio A, Rimm EB, Giovannucci E (1996). A prospective study of social networks in relation to total mortality and cardiovascular disease in men in the USA.. J Epidemiol Community Health.

[105] Keller BK, Magnuson TM, Cernin PA, Stoner JA, Potter JF (2003). The significance of social network in a geriatric assessment population.. Aging Clin Exp Res.

[106] Kiely DK, Simon SE, Jones RN, Morris JN (2000). The protective effect of social engagement on mortality in long-term care.. J Am Geriatr Soc.

[107] Kimmel PL, Peterson RA, Weihs KL, Shidler N, Simmens SJ (2000). Dyadic relationship conflict, gender, and mortality in Urban hemodialysis patients.. J Am Soc Nephrol.

[108] Korten AE, Jorm AF, Jaio Z, Letenneur L, Jacomb PA (1999). Health, cognitive, and psychosocial factors as predictors of mortality in an elderly community sample.. J Epidemiol Community Health.

[109] Krause N (1997). Received support, anticipated support, social class, and mortality.. Res Aging.

[110] Krause N (2006). Church-based social support and mortality.. J Gerontol.

[111] Kroenke CH, Kubzansky LD, Schernhammer ES, Holmes MD, Kawachi I (2006). Social networks, social support, and survival after breast cancer diagnosis.. J Clin Oncol.

[112] La Cour P, Avlund K, Schultz-Larsen K (2005). Religion and survival in a secular region. A twenty year follow-up of 734 Danish adults born in 1914.. Soc Sci Med.

[113] Lee M, Rotheram-Borus MJ (2001). Challenges associated with increased survival among parents living with HIV.. Am J Public Health.

[114] Lehto US, Ojanen M, Dyba T, Aromaa A, Kellokumpu-Lehtinen P (2006). Baseline psychosocial predictors of survival in localized breast cancer.. Br J Cancer.

[115] Lennartsson C, Silverstein M (2001). Does engagement with life enhance survival of elderly people in Sweden? The role of social and leisure activities.. J Gerentol.

[116] Ljungquist B, Berg S, Steen B (1995). Prediction of survival in 70-year olds.. Arch Gerontol Geriatr.

[117] Lund R, Due P, Modvig J, Holstein BE, Damsgaard MT (2002). Cohabitation and marital status as predictors of mortality–an eight year follow-up study.. Soc Sci Med.

[118] Lund R, Modvig J, Due P, Holstein BE (2000). Stability and change in structural social relations as predictor or mortality among elderly women and men.. Eur J Epidemiol.

[119] Lyyra T, Heikkinen R (2006). Perceived social support and mortality in older people.. J Gerontol.

[120] Maier D, Smith J (1999). Psychological predictors of mortality in old age.. J Gerontol: Series B: Psychol Scis & Social Sciences.

[121] Malmstrom M, Johansson S, Sundquist J (2001). A hierarchical analysis of long-term illness and mortality in socially deprived areas.. Soc Sci Med.

[122] McClellan WM, Stanwyck DJ, Anson CA (1993). Social support and subsequent mortality among patients with end-stage renal disease.. J Am Soc Nephrol.

[123] Merlo J, Ostergren P, Mansson N, Hanson BS, Ranstam J (2000). Mortality in elderly men with low psychosocial coping resources using anxiolytic-hypnotic drugs.. 1403–4948.

[124] Mertens JR, Moos RH, Brennan PL (1996). Alcohol consumption, life context, and coping predict mortality among late-middle-aged drinkers and former drinkers.. Alcohol Clin Exp Res.

[125] Morris PLP, Robinson RG, Andrzejewski P, Samuels J, Price TR (1993). Association of depression with 10-year post stroke mortality.. Am J Psychiatry.

[126] Murata C, Kondo T, Hori Y, Miyao D, Tamakoshi K (2005). Effects of social relationships on mortality among the elderly in a Japanese rural area: An 88-month follow-up study.. J Epidemiol.

[127] Murberg TA, Bru E (2001). Social relationships and mortality in patients with congestive heart failure.. J Psychosom Res.

[128] Musick MA, House JS, Williams DR (2004). Attendance at religious services and mortality in a national sample.. J Health Soc Behav.

[129] Nakanishi N, Tatara K (2000). Correlates and prognosis in relation to participation in social activities among older people living in a community in Osaka, Japan.. Journal of Clinical Geropsychology.

[130] Nordentoft M, Breum L, Munck LK, Nordestgaard AG, Hunding A, Bjaeldager PAL (1993). High mortality by natural and unnatural causes: A 10 year follow up study of patients admitted to a poisoning treatment centre after suicide attempts.. Br Med J.

[131] Olsen RB, Olsen J, Gunner-Svensson F, Waldstrom B (1991). Social networks and longevity: A 14 year follow-up study among elderly in Denmark.. Soc Sci Med.

[132] Oman D, Reed D (1998). Religion and mortality among the community-dwelling elderly.. Am J Public Health.

[133] Orrell M, Butler R, Bebbington P (2000). Social factors and the outcome of dementia.. Int J Geriatr Psychiatry.

[134] Orth-Gomer K, Johnson JV (1987). Social network interaction and mortality.. J Chronic Dis.

[135] Orth-Gomer K, Unden AL (1990). Type A behavior, social support, and coronary risk: Interaction and significance for mortality in cardiac patients.. Psychosom Med.

[136] Ostbye T, Krause KM, Norton MC, Tschanz J, Sanders L (2006). Cache County Investigators, Ten dimensions of health and their relationships with overall self-reported health and survival in a predominately religiously active elderly population: The Cache County memory study.. J Am Geriatr Soc.

[137] Oxman TE, Freeman DH, Manheimer ED (1995). Lack of social participation or religious strength and comfort as risk factors for death after cardiac surgery in the elderly.. Psychosom Med.

[138] Parkerson GR, Gutman RA (2000). Health-related quality of life predictors of survival and hospital utilization.. Health Care Financ Rev.

[139] Pennix BWJH, Tilburg T, Kriegsman DMW, Deeg DJH (1997). Effects of social support and personal coping resources on mortality in older age: The longitudinal aging study Amsterdam.. Am J Epidemiol.

[140] Rasulo D, Christensen K, Tomassini C (2005). The influence of social relations on mortality in later life: A study on elderly Danish twins.. Gerontologist.

[141] Reuben DB, Rubenstein LV, Hirsch SH, Hays RD (1992). Value of functional status as a predictor of mortality: Results of a prospective study.. Am J Med.

[142] Reynolds P, Boyd PT, Blacklow RS, Jackson JS, Greenberg RS (1994). The relationship between social ties and survival among Black and White breast cancer patients.. Cancer Epidemiol Biomarkers Prev 1055–9965.

[143] Rodriguez-Artalejo F, Guallar-Castillon P, Herrera MC, Otero CM, Chiva MO (2006). Social network as a predictor of hospital readmission and mortality among older patients with heart failure.. J Card Fail.

[144] Rosengren A, Orth-Gomer K, Wilhelmsen L (1998). Socioeconomic differences in health indices, social networks and mortality among Swedish men: A study of men born in 1933.. Scand J Soc Med.

[145] Roy AW, FitzGibbon PA, Haug MM (1996). Social support, household composition, and health behaviors as risk factors for four-year mortality in an urban elderly cohort.. J App Gerontol.

[146] Rozzini R, Bianchetti A, Franzoni S, Zanettie O, Trabucchi M (1991). Social, functional, and health status influences on mortality: Consideration of a multidimensional inquiry in a large elderly population.. J Cross Cult Gerontol.

[147] Ruberman W, Weinblatt E, Goldberg JD, Chaudhary BS (1984). Psychosocial influences on mortality after myocardial infarction.. N Engl J Med.

[148] Rutledge T, Matthews K, Lui L, Stone KL, Cauley JA (2003). Social networks and marital status predict mortality in older women: Prospective evidence from the Study of Osteoporotic Fractures (SOF).. Psychosom Med.

[149] Rutledge T, Reis SE, Olson M, Owens J, Kelsey SF (2004). National Heart, Lung, and Blood Institute, Social networks are associated with lower mortality rates among women with suspected coronary disease: the National Heart, Lung, and Blood Institute-sponsored Women's Ischemia Syndrome Evaluation Study.. Psychosom Med.

[150] Saito-Nakaya K, Nakaya N, Fujimori M, Akizuki N, Yoshikawa E, Kobayakawa M, Nagai K, Nishiwaki N, Tsubono Y, Uchitomi Y (2006). Marital status, social support and survival after curative resection in non-small-cell lung cancer.. Cancer Sci.

[151] Schoenbach VJ, Kaplan BH, Fredman L, Kleinbaum DG (1986). Social ties and mortality in Evans County, Georgia.. Am J Epidemiol.

[152] Seeman T, Berkman L, Kohout F, Lacroix A, Glynn R (1993). Intercommunity variations in the association between social ties and mortality in the elderly: A comparative analysis of three communities.. Eur Psychiatry.

[153] Shahatahmasebi S, Davies R, Wenger GC (1992). A longitudinal analysis of factors related to survival in old age.. Gerontologist.

[154] Shmotkin D, Blumstein T, Modan B (2003). Beyond keeping active: Concomitants of being a volunteer in old-old age.. Psychol Aging.

[155] Shye D, Mullooly JP, Freeborn DK, Pope CR (1995). Gender differences in the relationship between social network support and mortality: A longitudinal study of an elderly cohort.. Soc Sci Med.

[156] Silverstein M, Bengtson VL (1991). Do close parent-child relations reduce the mortality risk of older parents?. J Health Soc Behav.

[157] Soler-Vila H, Kasl SV, Jones BA (2003). Prognostic significance of psychosocial factors in African-American and White breast cancer patients: A population based study.. Cancer.

[158] Stavraky KM, Donner AP, Kincade JE, Stewart MA (1988). The effect of psychosocial factors on lung cancer mortality at one year.. J Clin Epidemiol.

[159] Stek ML, Vinkers DJ, Gussekloo J, Beekman ATF, Van der Mast RC (2005). Is depression in old age fatal only when people feel lonely?. The Am J Psychiatry.

[160] Sturdy PM, Victor CR, Anderson HR, Bland JM, Butland BK (2002). Psychological, social and health behavior risk factors for deaths certified as asthma: A national case-control study.. Thorax.

[161] Sugisawa H, Liang J, Liu X (1994). Social networks, social support, and mortality among older people in Japan.. J Gerontol.

[162] Sun R, Liu Y (2006). Mortality of the oldest old in China: The role of social and solitary customary activities.. J Aging Health.

[163] Temkin-Greener H, Bajorska A, Peterson DR, Kunitz SJ, Gross D (2004). Social support and risk-adjusted mortality in a frail, older population.. Med Care.

[164] Thomas SA, Friedmann E, Wimbush F, Schron E (1997). Psychosocial factors and survival in the Cardiac Arrhythmia Suppression Trial (CAST): A reexamination.. Am J Crit Care.

[165] Tucker JS, Friedman HS, Wingard DL, Schwartz JE (1996). Marital history at midlife as a predictor of longevity: alternative explanations to the protective effects of marriage.. Health Psychol.

[166] Vaillant GE, Meyer SE, Mukaamal K, Soldz S (1998). Are social supports in late midlife a cause or a result of successful physical ageing?. Psychol Med.

[167] Vogt TM, Mulloolly DE, Ernst E, Pope CR, Hollis JF (1992). Social networks as predictors of ischemic heart disease, cancer, stroke, and hypertension: incidence, survival, and mortality.. J Clin Epidemiol.

[168] Walter-Ginzburg A, Blumstein T, Chetrit A, Modan B (2002). Social factors and mortality in old-old in Israel: The CALAS study.. J Gerontol.

[169] Waxler-Morrison N, Hislop G, Mears B, Kan L (1991). Effects of social relationships on survival for women with breast cancer: A prospective study.. Soc Sci Med.

[170] Weihs KL, Simmens SJ, Mizrahi J, Enright TM, Hunt ME (2005). Dependable social relationships predict overall survival in stages II and III breast carcinoma patients.. J Psychosom Res.

[171] Welin C, Lappas G, Wilhelmsen L (2000). Independent importance of psychosocial factors for prognosis after myocardial infarction.. J Intern Med.

[172] Welin L, Larsson B, Svardsudd K, Tibblin B, Tibblin G (1992). Social network and activities in relation to mortality from cardiovascular diseases, cancer, and other causes: A 12 year follow up of the Study of Men Born in 1913 and 1923.. Journal of Epidemiology and Community Health.

[173] Wilkins K (2003). Social support and mortality in seniors.. Health Rep.

[174] Woloshin S, Schwartz LM, Tosteson ANA, Chang CH, Wright B (1997). Perceived adequacy of tangible social support and health outcomes in patients with coronary artery disease.. J Gen Intern Med.

[175] Yasuda N, Zimmerman SI, Hawkes W, Fredman L, Hebel JR (1997). Relation of social network characteristics to 5-year mortality among young-old versus old-old White women in an urban community.. Am J Epidemiol.

[176] Zuckerman DM, Kasl SV, Ostfeld AM (1984). Psychosocial predictors of mortality among the elderly poor.. Am J Epidemiol.

[177] Rosenthal, The file drawer problem and tolerance for null results.. Psychol Bull.

[178] Duval S, Tweedie R (2000). A non-parametric “trim and fill” method of accounting for publication bias in meta-analysis.. J Am Stat Assoc.

[179] Duval S, Tweedie R (2000). Trim and fill: A simple funnel-plot based method of testing and adjusting for publication bias in meta-analysis.. Biometrics.

[180] Peters JL, Sutton AJ, Jones DR, Abrams KR, Rushton L (2006). Comparison of two methods to detect publication bias in meta-analysis.. JAMA.

[181] Peters J, Sutton A, Jones D, Abrams K, Rushton L Contour-enhanced meta-analysis funnel plots help distinguish publication bias from other causes of asymmetry.. Journal Of Clinical Epidemiology.

[182] Uchino BN (2006). Social support and health: a review of physiological processes potentially underlying links to disease outcomes.. J Behav Med.

[183] Samet JM (1990). The 1990 Report of the Surgeon General: The Health Benefits of Smoking Cessation.. Am Rev Respir Dis.

[184] Friedman HS, Tucker JS, Schwartz JE, Tomlinson-Keasey C, Martin LR (1995). Psychosocial and behavioral predictors of longevity: The aging and death of the ‘Termites’.. Am Psychol.

[185] Tucker JS, Friedman HS, Wingard DL, Schwartz JE (1996). Marital history at midlife as a predictor of longevity: Alternative explanations to the protective effect of marriage.. Health Psychol.

[186] Coyne JC, Rohrbaugh MJ, Shoham V, Sonnega JS, Nicklas JM (2001). Prognostic importance of marital quality for survival of congestive heart failure.. Am J Cardiol.

[187] Eaker ED, Sullivan LM, Kelly-Hayes M, D'Agostino RB, Benjamin EJ (2007). Marital status, marital strain, and risk of coronary heart disease or total mortality: the Framingham Offspring Study.. Psychosom Med.

[188] Rothman KJ, Greenland S, Lash TL (2008). Modern Epidemiology.

[189] Uchino BN, Cacioppo JT, Kiecolt-Glaser JK (1996). The relationship between social support and physiological processes: A review with emphasis on underlying mechanisms and implications for health.. Psychol Bull.

[190] Rozanski A, Blumenthal JA, Kaplan J (1999). Impact of psychological factors on the pathogenesis of cardiovascular disease and implications for therapy.. Circulation.

[191] Cohen S (1988). Psychosocial models of the role of social support in the etiology of physical disease.. Health Psychol.

[192] Uchino BN, Holt-Lunstad J, Uno D, Campo R, Reblin M (2007). The Social Neuroscience of Relationships: An Examination of Health-Relevant Pathways. Social neuroscience: Integrating biological and psychological explanations of social behavior.

[193] Uchino BN, Uno D, Holt-Lunstad J (1999). Social support, physiological processes, and health.. Curr Dir Psychol Sci.

[194] Lutgendorf SK, Sood AK, Anderson B, McGinn S, Maiseri H (2005). Social support, psychological distress, and natural killer cell activity in ovarian cancer.. J Clin Oncol.

[195] Miyazaki T, Ishikawa T, Nakata A, Sakurai T, Miki A (2005). Association between perceived social support and Th1 dominance.. Biol Psychol.

[196] Moynihan JA, Larson MR, Treanor J, Duberstein PR, Power A (2004). Psychosocial factors and the response to influenza vaccination in older adults.. Psychosom Med.

[197] Cohen S, Doyle WJ, Skoner DP, Rabin BS, Gwaltney JM (1997). Social ties and susceptibility to the common cold.. JAMA.

[198] Kiecolt-Glaser JK, Loving TJ, Stowell JR, Malarkey WB, Lemeshow S (2005). Hostile marital interactions, proinflammatory cytokine production, and wound healing.. Arch Gen Psychiatry.

[199] Spiegel D, Bloom JR, Kraemer H, Gottheil E (1989). Psychological support for cancer patients.. Lancet.

[200] Altman DG (1995). Sustaining interventions in community systems: On the relationship between researchers and communities.. Health Psychology.

[201] Spitz RA (1945). Hospitalism: An inquiry into the genesis of psychiatric conditions in early childhood.. Psychoanalytic Study of the Child.

[202] Bowlby J (1951). Maternal care and mental health.

[203] Provence SA, Lipton RC (1962). Infants in institutions.

[204] UNICEF (1997). Children at risk in Central and Eastern Europe: Perils and promises.

[205] Shavelle RM, Paculdo DR, Strauss DJ, Kush SJ (2008). Smoking habit and mortality: a meta-analysis.. J Insur Med.

[206] Critchley JA, Capewell S (2003). Mortality risk reduction associated with smoking cessation in patients with coronary heart disease: a systematic review.. JAMA.

[207] Holman CD, English DR, Milne E, Winter MG (1996). Meta-analysis of alcohol and all-cause mortality: a validation of NHMRC recommendations.. Med J Aust.

[208] Fine MJ, Smith MA, Carson CA, Meffe F, Sankey SS (1994). Efficacy of pneumococcal vaccination in adults. A meta-analysis of randomized controlled trials.. Arch Intern Med.

[209] Taylor RS, Brown A, Ebrahim S, Jolliffe J, Noorani H (2004). Exercise-based rehabilitation for patients with coronary heart disease: systematic review and meta-analysis of randomized controlled trials.. Am J Med.

[210] Katzmarzyk PT, Janssen I, Ardern CI (2003). Physical inactivity, excess adiposity and premature mortality.. Obes Rev.

[211] Insua JT, Sacks HS, Lau TS, Lau J, Reitman D (1994). Drug treatment of hypertension in the elderly: a meta-analysis.. Ann Intern Med.

[212] Schwartz J (1994). Air pollution and daily mortality: a review and meta analysis.. Environ Res.

[213] Barrera M, Sandler I, Ramsay T (1981). Preliminary development of a scale of social support: Studies on college students.. Am J Commun Psychol.

[214] Dunkel-Schetter C, Folkman S, Lazarus R (1987). Correlates of social support receipt.. J Pers Soc Psychol.

[215] Dunkel-Schetter C, Feinstein L, Call J (1987). UCLA Social Support Inventory..

[216] Vaux A, Riedel S, Stewart D (1987). Models of social support: The social support behaviors (SS-B) scale.. Am J Commun Psychol.

[217] Seeman TE, Berkman LF (1988). Structural characteristics of social support networks and their relationship with social support in the elderly: Who provides support.. Soc Sci Med.

[218] Hanson BS, Ostergren P-O, Elmstahl S, Isacsson S-O, Ranstam J (1997). Reliability and validity assessments of measures of social networks, social support and control — results from the Malmo Shoulder and Neck Study.. Scand J Soc Med.

[219] Sarason IG, Levine HM, Basham RB, Sarason BR (1983). Assessing social support: The social support questionnaire.. J Pers Soc Psychol.

[220] Cohen S, Hoberman HM (1983). Positive events and social support as buffers of life change stress.. J Appl Soc Psychol.

[221] De Jong-Gierveld J, Kamphuis F (1985). The development of a Rasch-type loneliness scale.. Appl Psych Meas.

[222] Russell D, Peplau LA, Cutrona CE (1980). The revised UCLA Loneliness Scale: Concurrent and discriminant validity evidence.. J Pers Soc Psychol.

[223] Kahn RL, Antonucci TC, Baltes PB, Brim 0 (1980). Convoys over the life course: Attachment, roles and social support.. Life span development and behavior.

[224] Hirsch BJ (1979). Psychological dimensions of social networks: A multi-method analysis.. Am J Commun Psychol.

[225] Hanson BS, Ostergren P-O, Elmstahl S, Isacsson S-O, Ranstam J (1997). Reliability and validity assessments of measures of social networks, social support and control — results from the Malmo Shoulder and Neck Study.. Scand J Soc Med.

[226] Cohen S, Greene AL, Cummings M, Karraker KH (1991). Social supports and physical health.. Life-Span Developmental Psychology: Perspectives on Stress and Coping.

[227] Cohen S, Doyle WJ, Skoner DP, Rabin BS, Gwaltney JM (1997). Social ties and susceptibility to the common cold.. JAMA.

[228] Kahn RL, Antonucci TC (1984). Social supports of the elderly: Family, friends, professionals (Refort No. AGO 01632).

[229] Donald CA, Ware JE (1984). The measurement of social support.. Res Commun Mental Health.

