# Supplementary material for: Social Relationships and Mortality Risk: A Meta-analytic Review
Source: PLoS Med. 2010 Jul 27;7(7):e1000316. doi: 10.1371/journal.pmed.1000316 (PMC2910600; doi:10.1371/journal.pmed.1000316)
Supplement: Alternative Language Abstract S1 — Abstract translated into Japanese by Hideko Cannell. (0.02 MB DOC) [file pmed.1000316.s001.doc]

要旨

背景：個人の社会関係の質と量は、精神衛生だけではなく、罹患率と死亡率にも関連している。

目的：どの社会関係が死亡リスクに影響を及ぼし、社会関係のどの側面が最も予見性が高く、そしてどの因子によってリスクが緩和されるのかを明らかにするためにこのメタ分析総説を実施した。

データ抽出：死因、初期の健康状態、既往の疾病など参加者の特徴に関するデータに加え経過観察期間の長さと社会関係の評価タイプなど調査特徴におけるデータを抽出した。

結果：148の調査（参加者308,849人）の結果から、変量効果量の重み付け平均のオッズ比は1.50 (95%信頼区間1.42 - 1.59) となり、社会関係が強い患者において生存率が50％高くなる可能性があることが示唆された。この所見は年齢、性別、初期の健康状態、死因、観察期間に関わらず一致してみられた。評価を行った社会指標のいずれのタイプにも有意差が見られた(*p* < .001)。つまり、社会的一体化の指標が複雑な場合には関連性が最も強く、(OR=1.91; 95%信頼区間 1.63 - 2.23)、

居住状況のバイナリ指標の場合に最も弱かった。（一人暮らしまたは複数でくらす）(OR=1.19; 95%信頼区間0.99 - 1.44)

結論：死亡の危険因子に対する社会関係の影響は、確立された死亡の危険因子と同程度である。
