# Supplementary material for: Social Relationships and Mortality Risk: A Meta-analytic Review
Source: PLoS Med. 2010 Jul 27;7(7):e1000316. doi: 10.1371/journal.pmed.1000316 (PMC2910600; doi:10.1371/journal.pmed.1000316)
Supplement: Alternative Language Abstract S2 — Abstract translated into Spanish by Rod Veas. (0.03 MB DOC) [file pmed.1000316.s002.doc]

**Resumen**

**Antecedentes**: La calidad y cantidad de lazos sociales de individuos ha sido vinculada no solamente a la salud mental pero también a la propensión a enfermedad y mortalidad.

**Objetivos:** Este estudio meta-analítico fue llevado a cabo para determinar el grado de influencia que tienen los lazos sociales sobre el riesgo de mortalidad, cuáles aspectos de los lazos sociales son los más predictivos, y qué factores pueden moderar el riesgo.

**Extracción de Data:** Data fue extraída en cuanto a varias características de participantes, incluyendo causa de mortalidad, estado de salud inicial, y condiciones médicas pre-existentes. Además se recolectó data en cuanto a las características del estudio,  incluyendo plazo de seguimiento y tipo de medidas de  lazos sociales.
**Resultados:** A través de 148 estudios (308,849 participantes), el efecto aleatorio ponderado del tamaño de efecto fue *OR* = 1.50 (IC de 95%, 1.42 a 1.59), indicando un incremento de 50% en la probabilidad de supervivencia para participantes con  lazos sociales más fuertes. Este resultado fue consistente a través de edad, sexo, estado de salud inicial, causa de muerte y plazo de seguimiento. Diferencias significativas fueron encontradas a través de tipo de medida social evaluada (p < .001); la asociación fue más fuerte para medidas complejas de integración social  (OR=1.91; IC de 95%, 1.63 a 2.23) y más débil para indicadores binarios de estado de residencia (viviendo sólo versus con otros) (OR=1.19; IC de 95%, 0.99 a 1.44).

**Conclusiones:** La influencia de lazos sociales sobre el riesgo de mortalidad es comparable a factores de riesgo de mortalidad ya bien establecidos.
