# Supplementary material for: Social Relationships and Mortality Risk: A Meta-analytic Review
Source: PLoS Med. 2010 Jul 27;7(7):e1000316. doi: 10.1371/journal.pmed.1000316 (PMC2910600; doi:10.1371/journal.pmed.1000316)
Supplement: Text S2 — Review protocol. (0.05 MB DOC) [file pmed.1000316.s004.doc]

**REVIEW PROTOCOL**

1. Objective

Evaluating the odds of survival/mortality as a function of previously measured social relationships.

1. Endpoint

Participant mortality status (alive/dead).

1. Selection criteria

To be included in the meta-analysis, manuscripts must have fulfilled the following criteria:

- 1. Methodological criteria

All quantitative research designs included except for:

- - Case studies
  - Single-N designs
  - Aggregate-level data (i.e., census data) not directly linked to individuals
  1. Cause of mortality

All mortality outcomes were included except for:

- - Suicide
  - Injury
  - Mortality combined with other indicators (i.e., morbidity)
  1. Type of social relationship measured

All types of social relationships measured were included except for:

- - Those provided within the context of the study (i.e., support group)
  - Non-human support
  - Social support provided to others (i.e., giving support to others)
  - Marital status, if it is the only indicator reported (otherwise, included as one of several measures)

1. Literature search

Ten electronic databases were searched:

Medline

HealthSTAR

Mental Health Abstracts

PsycINFO

Dissertation Abstracts

Academic Search Premier

ERIC

Family & Society Studies Worldwide.

Social Sciences Abstracts

Sociological Abstracts via SocioFile

Databases searched included the range of January 1900 to January 2007.

To reduce inadvertent omissions, we searched databases yielding the most citations (Medline, PsycINFO) two additional times.

Database searches combined (via Boolean “AND”) sets of search terms on the following three constructs: social relationships, mortality, and quantitative data.

*Social Relationships*

("social network*" or "social support*" or "social integration" or "social participation" or "social cohesion" or "interpersonal support" or "interpersonal cohesion" or "social well-being" or "interpersonal integration" or "social relationship*" or "social capital" or "social isolation" or "social interaction" or "social influenc*" or "social ties" or "marital status" or “social contact” or “living alone” or “social isolation” or “social activit*” or married or marriage or “marital disruption” or “Marital status” or divorced or widow* or “marital history” or “social resource*” or “social bonds” or “social connection” or intimacy or “perceived support” or “social provision” or “sense of community” or “support* behavior*” or “social communit*” or lonel* or DE "Friendship" or DE "Significant Others" or DE "Social Interaction" or DE "Support Groups" or DE "Family Relations" or DE "Interpersonal Relationships" or DE "Marital Relations" or DE "Relationship Satisfaction" or DE "Cohabitation" or DE "Family Members" or DE "Family" or DE "Couples" or DE "Spouses" or MM "Social Networks" or DE "Social Capital" or DE "Social Groups" or MM "Social Support" or DE "Interpersonal Interaction" or DE "Assistance Social Behavior" or DE "Employee Interaction" or DE "Group Participation" or DE "Peer Relations" or DE "Intimacy")

*Mortality*

(mortality or death or deceased or died or dead or "remain* alive" or fatal* or dying or "loss of life" or decease*)

*Quantitative Data*

(outcome or data or subjects or experiment or results or empirical or quantitative or longitudinal or predictive or prospective or measure* or statistic* or study or research or survey or finding* or analy* or method or participants or "risk ratio" or "hazard ratio" or regression or number* or effects or predictor or baseline or evaluation or recruited or screened or “follow* up” or tracked or re-evaluat* or re-assess* or re-test* or “more likely” or “less likely” or hazards or odds or rate or “over time” or “years later”)

Limits: Written in English.

We manually examined the reference sections of past reviews and of studies meeting the inclusion criteria to locate articles not identified in the database searches.

To obtain additional manuscript, we sent solicitation letters to authors who had published three or more articles on the topic.

1. Data extraction

The following information was extracted and entered *independently* into databases by two teams of two coders:

1. Country of data collection
2. Setting of initial data collection
3. Percent female
4. Percent married
5. Mean Age of participants at first assessment
6. Length of follow-up (in years)
7. Research Design type
8. Cause of Mortality
9. Percent Smokers in Sample
10. Percent Physically Active in Sample (vs. sedentary or inactive)
11. Initial Health Status of Sample
12. Pre-existing health condition (at intake)
13. Odds ratio
14. Log odds ratio (natural log odds ratio)
15. Standard Error of effect size
16. Total sample size that *this effect size* is based on
17. Percent deceased (by follow-up)
18. How Social Support was assessed
19. Statistical controls for the effect size

When multiple effect sizes were reported within a study at the same point in time (e.g., across different measures of social support), we averaged the several values (weighted by N) to avoid violating the assumption of independent samples. When multiple reports contained data from the same participants (publications of the same database), we selected the report containing the whole sample and eliminated reports of sub-samples. When multiple reports contained the same whole sample, we selected the one with the longest follow-up duration. When multiple reports with the same whole sample were of the same duration, we selected the one reporting the greatest number of measures of social support. In cases where multiple effect sizes were reported across different levels of social support (i.e., high vs. medium, medium vs. low), we extracted the value with the greatest contrast (i.e., high vs. low). When a study contained multiple effect sizes across time, we extracted the data from the longest follow-up period. If a study used statistical controls in calculating an effect size, we extracted the data from the model utilizing the fewest statistical controls (and we recorded the type and number of covariates used within each study to run post hoc comparative analyses). We coded the research design used rather than estimate risk of individual study bias.

1. Statistical analyses

Effect sizes reported within studies were transformed to the metric of lnOR for analyses and then transformed back to OR for purposes of interpretation. I^2^ describes the percentage of variability in point estimates that is due to heterogeneity rather than sampling error.

Analyses were performed using Comprehensive Meta-Analysis software and SPSS macros developed by Lipsey and Wilson.

Random-effect models were used for all analyses. A random effects approach yields results that generalize beyond the sample of studies actually reviewed. The assumptions made in this meta-analysis clearly warrant this method: The belief that certain variables serve as moderators of the observed association between symptoms of depression and religious involvement implies that the studies reviewed will estimate different population effect sizes. Random effects models take such between-studies variation into account, whereas fixed effects models do not.

A contour enhanced funnel plot was used to investigate publication bias.
